# Integrative omics analyses of the ligninolytic *Rhodosporidium fluviale* LM-2 disclose catabolic pathways for biobased chemical production

**DOI:** 10.1186/s13068-022-02251-6

**Published:** 2023-01-09

**Authors:** Nathália Vilela, Geizecler Tomazetto, Thiago Augusto Gonçalves, Victoria Sodré, Gabriela Felix Persinoti, Eduardo Cruz Moraes, Arthur Henrique Cavalcante de Oliveira, Stephanie Nemesio da Silva, Taícia Pacheco Fill, André Damasio, Fabio Marcio Squina

**Affiliations:** 1 Programa de Processos Tecnológicos e Ambientais, University of Sorocaba (UNISO), Sorocaba, Brazil; 2 Department of Biochemistry and Tissue Biology, Institute of Biology, University of Campinas (UNICAMP), Campinas, Brazil; 3 Department of Biological and Chemical Engineering (BCE), Aarhus University, 8200 Aarhus, Denmark; 4 Photobiocatalysis Unit—CPBL, and Biomass Transformation Lab—BTL, École Interfacultaire de Bioingénieurs, Université Libre de Bruxelles, Brussels, Belgium; 5 Department of Chemistry, University of Warwick, Coventry, UK; 6 Brazilian Biorenewables National Laboratory (LNBR), Brazilian Center for Research in Energy and Materials (CNPEM), Campinas, Brazil; 7 Department of Chemistry, Faculty of Philosophy Sciences and Letters of Ribeirão Preto, University of São Paulo, Ribeirão Preto, SP Brazil; 8 Laboratory of Biology Chemical Microbial (LaBioQuiMi), Institute of Chemistry, University of Campinas (UNICAMP), Campinas, Brazil

**Keywords:** Lignin valorization, Ferulic acid, 4-Vinyl guaiacol, Vanillin, *Rhodosporidium fluviale*

## Abstract

**Background:**

Lignin is an attractive alternative for producing biobased chemicals. It is the second major component of the plant cell wall and is an abundant natural source of aromatic compounds. Lignin degradation using microbial oxidative enzymes that depolymerize lignin and catabolize aromatic compounds into central metabolic intermediates is a promising strategy for lignin valorization. However, the intrinsic heterogeneity and recalcitrance of lignin severely hinder its biocatalytic conversion. In this context, examining microbial degradation systems can provide a fundamental understanding of the pathways and enzymes that are useful for lignin conversion into biotechnologically relevant compounds.

**Results:**

Lignin-degrading catabolism of a novel *Rhodosporidium fluviale* strain LM-2 was characterized using multi-omic strategies. This strain was previously isolated from a ligninolytic microbial consortium and presents a set of enzymes related to lignin depolymerization and aromatic compound catabolism. Furthermore, two catabolic routes for producing 4-vinyl guaiacol and vanillin were identified in *R. fluviale* LM-2.

**Conclusions:**

The multi-omic analysis of *R. fluviale* LM-2, the first for this species, elucidated a repertoire of genes, transcripts, and secreted proteins involved in lignin degradation. This study expands the understanding of ligninolytic metabolism in a non-conventional yeast, which has the potential for future genetic manipulation. Moreover, this work unveiled critical pathways and enzymes that can be exported to other systems, including model organisms, for lignin valorization.

**Supplementary Information:**

The online version contains supplementary material available at 10.1186/s13068-022-02251-6.

## Background

Lignocellulosic biomass is the most abundant and low-cost source of fermentable sugars and building blocks for the production of biofuels and value-added chemicals, which makes it an attractive alternative to fossil fuels [1]. It is primarily composed of cellulose, hemicellulose, and lignin. While cellulose and/or hemicellulose can be utilized to produce biofuels and chemicals, the use of lignin has been limited to energy supply.

Lignin is the second major component of plant cell walls, imparting structural stability to plant tissues and fibers, and forming a barrier against microbial infections [2]. It consists of phenylpropanoid monomeric guaiacyl (G), *p*-hydroxyphenyl (H), and syringyl (S) units randomly interconnected mainly by β-O-4 aryl ether bonds [3]. Considering that 70 million tons of lignin is extracted annually during pulping operations [4], lignin is an abundant natural source of aromatic compounds*.* However, lignin valorization requires efficient methods for the degradation and conversion of complex mixtures of lignin-derived aromatic compounds into bioproducts. Some microorganisms have been described as being capable of bioconverting lignin into value-added compounds, such as vanillin [5], polyester precursors [6, 7], and even nylon [8], indicating their usefulness for lignin valorization.

In nature, lignin bioprocessing occurs through symbiotic activity, involving white-rot fungi and certain bacterial species, and consists of two steps [9, 10]: (i) lignin depolymerization and (ii) aromatic ring fission. In the first step, lignin macropolymer degraders, such as *Phanerochaete chrysosporium*, *Schizophyllum commune*, and *Pseudomonas putida*, secrete an enzymatic cocktail consisting of oxidative enzymes (e.g., laccases and peroxidases) and accessory enzymes to cleave the linkages between S, G, and H units. Laccases and class II heme-dependent lignin-modifying peroxidases (e.g., lignin peroxidase (LiP) and manganese peroxidase (MnP)) act directly on lignin and lignin fragments [1, 11]. Auxiliary enzymes such as glyoxal oxidases (GLOX) support lignin degradation by producing H_2_O_2_ [12], the main substrate for peroxidase reactions (13). In addition to secreting extracellular enzymes, certain microorganisms produce β-etherase and cytochrome P450 (CYP), which cleave β-O-4 bonds and demethylate intracellular lignin fragments, respectively [14, 15].

In the second step, bacteria, fungi, and yeasts assimilate aromatic compounds through funneling pathways, converting the molecules into key central metabolic intermediates or target compounds [10, 16]. Despite the diverse array of oxidized fragments originating from lignin degradation, catabolism proceeds mainly via two major compounds, protocatechuate and catechol (both of which are cleaved to form central intermediates) [9, 16]. For instance, during the degradation of G-unit rich lignin, ferulic acid is converted into vanillin by feruloyl-CoA synthetase (FCS) and feruloyl-CoA hydratase-lyase (FCHL). Vanillin is then cleaved into protocatechuate (vanillin pathway), and the latter is converted into central intermediates (pyruvate, acetyl-CoA, and succinate) through three different pathways, namely the protocatechuate 4,5-cleavage, protocatechuate 2,3-cleavage, and β-ketoadipate pathways [10].

An alternative catabolic route has been described for ferulic acid bioconversion in *Rhodotorula rubra* [17], *Candida guilliermondii* [18], and *Cupriavidus* sp. B-8 [19]. In these microorganisms, ferulic acid is converted into 4-vinylguaiacol (4-VG) using the cofactor-free enzyme phenolic acid decarboxylase (PDC) [20]. Furthermore, 4-VG can be converted to vanillin and subsequently to protocatechuate [21]. Both ferulic acid catabolism pathways have potential biotechnological applications since vanillin and 4-VG are considered value-added bioproducts, and are widely used in the food and cosmetics industries [5, 22].

Although lignin degradation is mainly attributed to white-rot fungi and bacteria, oleaginous yeasts have been reported to be capable of degrading lignin and further assimilating the lignin-derived aromatic compounds. *Rhodosporidium* sp. can modify wheat straw and Sarkanda grass [23] and consume *p*-coumaric, *p*-hydroxybenzoic, and ferulic acids [24, 25]. Additionally, species of this genus have high tolerance toward inhibitors generated during the pretreatment of lignocellulosic biomass [26]. Even though some model microorganisms such as *Saccharomyces cerevisiae* and *Escherichia coli,* have been engineered for lignin valorization [27, 28], non-model microbes have also been continually characterized to unveil novel ligninolytic pathways with biotechnological relevance. For instance, *Rhodosporidium toruloides* is genetically and physiologically well characterized, providing vital knowledge for further strain engineering [29].

**Fig. 1:**
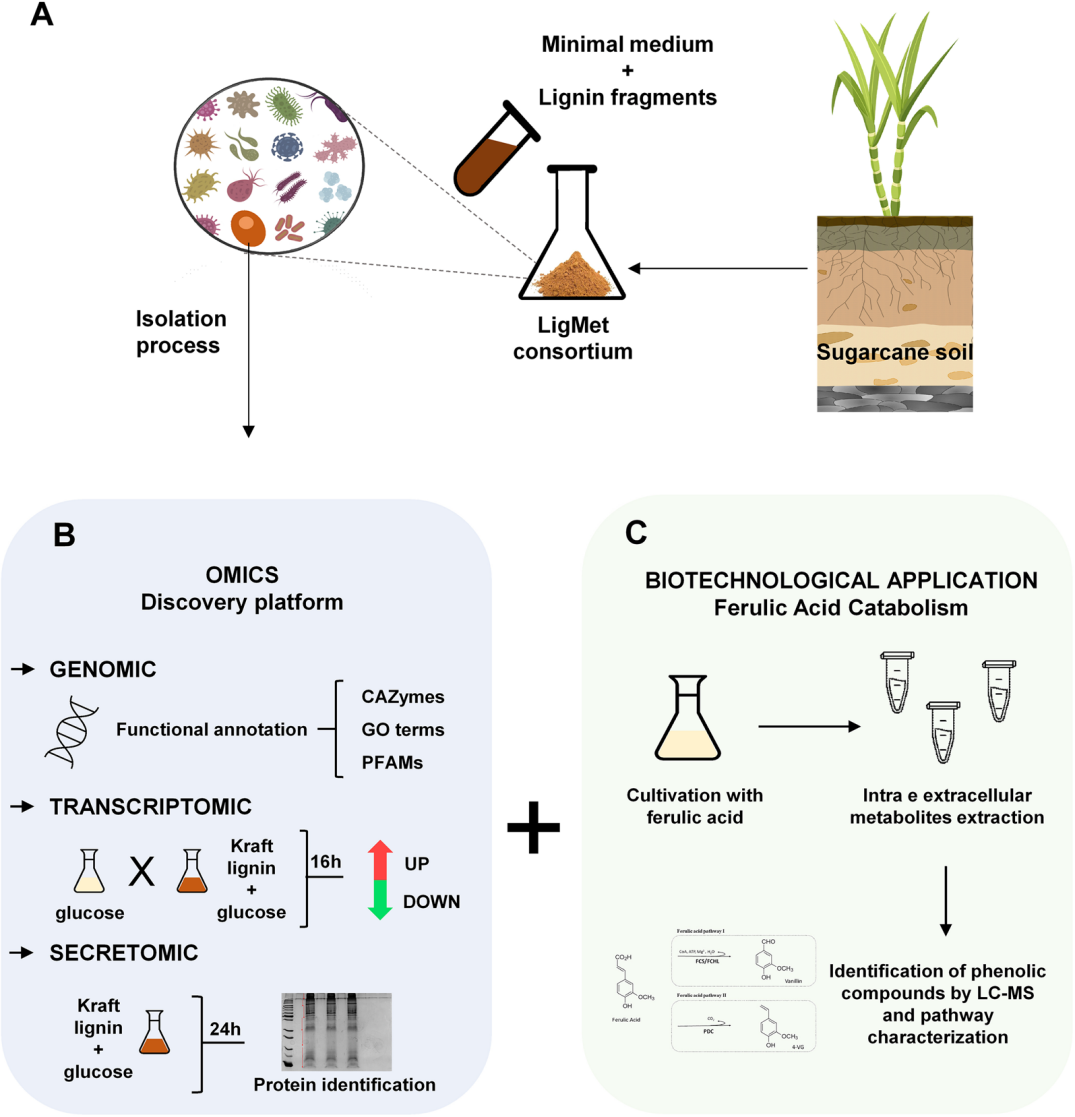
Schematic representation of the methods used in the study. **A** *R. fluviale* LM-2 isolated from the lignin-degrading consortium LigMet [30]. B) Omic approaches were used to characterize the genome, gene expressions, and secreted proteins related to lignin degradation. C) *R. fluviale* LM-2 cells were cultivated in a ferulic acid-containing medium to identify the active catabolic pathways for this compound

In this context, a combination of omics approaches was used to determine the genetic potential and physiology of a novel *Rhodosporidium fluviale* strain LM-2 isolated from a lignin-degrading microbial consortium [30] (Fig. 1). Genomic analysis revealed several genes involved in lignin degradation and aromatic catabolism, and transcriptomic and secretomic analyses elucidated the metabolism of yeast grown in lignin-containing medium. To exploit the biotechnological potential of *R. fluviale* for the production of compounds of interest, this novel strain was cultivated in media containing ferulic acid. Afterwards, the resulting metabolites were identified by UHPLC–MS/MS to determine possible pathways for ferulic acid catabolism in this yeast. Combining these results and omics approaches, biocatalytic pathways that may be useful for lignin valorization strategies were identified in this novel yeast.

## Results

### Isolation and identification

In a previous study, Moraes and collaborators (2018) isolated and identified several bacteria and yeasts in the LigMet microbial community, a lignin-degrading consortium developed by growing cultures on low-molecular-weight (LW) lignin [30]. For isolation of microorganisms, the culture broth from LigMet was diluted and plated on agar supplemented with LW lignin and high-molecular-weight (HW) lignin with glucose (HW + G) [30]. For the analysis described herein, the yeast strain LM-2 was selected, which was capable of growing on LW and HW + G plates (data not shown) and was highly tolerant to different kraft lignin concentrations (Additional file 1: Fig. S1).

To identify the LM-2, the ITS1 and ITS2 regions were sequenced, and a BLAST search was performed against the GenBank nucleotide (nonredundant) database. The analysis revealed that LM-2 shared 100% and 98% sequence identity with *R. fluviale* and *Rhodosporidium azoricum*, respectively (Table 1), and clustered together with *R. fluviale DMKU RK253* [31] based on phylogenetic tree construction using ITS regions (Additional file 2: Fig. S2). This indicated that LM-2 was a novel *R. fluviale* strain and was therefore named *R. fluviale* LM-2.

**Table 1:** Closest organism matches on GenBank nr (nonredundant) database

Gene	Closest match organism	Accession number	Identity (%)
ITS1/ITS2	*Rhodosporidium fluviale*^1^ DMKU-RK253	KT428589	100
	*Rhodosporidium fluviale*^1^ CBS 6568	NR_077089	100
	*Rhodosporidium fluviale*^1^ LEMI 369	JX512682	99.81
	*Rhodosporidium fluviale*^1^ UA2	FJ515206	99.81
	*Rhodosporidium fluviale*^1^ SJ42	FJ515184	99.81
	*Rhodosporidium azoricum*^2^ PYCC 4648	JN246556	99.23
	*Rhodosporidium azoricum*^2^ CBS 8949	NR_155729	98.07
D1/D2	*Rhodosporidium fluviale*^1^ HAI-Y-088	KC006468	100
	*Rhodosporidium fluviale*^1^ MB2021	KC798417	99.30
	*Rhodosporidium fluviale*^1^ HB92-2	KJ507301	99.83
	*Rhodosporidium fluviale*^1^ CBS6568	AY745719	99.65
	*Rhodosporidium azoricum*^2^ CBS 4648	DQ531944	99.65

These results were obtained using BLASTn search against hypervariable internal transcribed spacer ITS1/ITS2 sequences

^1^Homotypic synonym: *Rhodosporidiobolus fluvialis* [81]

^2^Homotypic synonym: *Rhodosporidiobolus azoricus* [81]

Physiological tests based on cell viability at different temperatures have been used to distinguish between *R. azoricum* and *R. fluviale* [32, 33]. *R. fluviale* can grow at both 30 and 37 ℃, whereas *R. azoricum* grows at 30 ℃ [32]. LM-2 showed the same temperature tolerance at 30 and 37 ℃ (Additional file 3: Fig. S3), corroborating the result of species determination by phylogenetic analyses.

### Genomic analysis and functional annotation

Illumina sequencing generated 9,839,814 paired-end, and 5,873,616 mated-pair reads (Additional file 7: Table S1). The assembled *R. fluviale* LM-2 genome was 50.7 Mb in length, distributed in 337 contigs with 60.15% G + C content, and a contig N50 length of 324 Kbp (Table 2). The assembly assessed by Benchmarking Universal Single-Copy Orthologous (BUSCO) revealed 235 (92.2%) completeness, and 130 (51%) complete and duplicated, 9 (3.5%) fragmented, and 11 (4.3%) missing BUSCO genes. Gene prediction identified 17,565 open reading frames (ORFs), with an average of 1589 bp per gene, most of which contained at least one intron.

**Table 2:** General features of the *R. fluviale* LM-2 genome

Features	Counts
Genome size	50.66 (Mb)
Number of contigs	337
Largest contig	1,011,807 (bp)
N50	324,989 (bp)
N75	185,817 (bp)
L50	48
L75	97
GC	60.5%
Genes	17,942
Protein coding genes	17,565
Nonprotein coding genes	377
tRNA	120
Average gene size	1,589 (bp)
Exons	111,773

Based on functional analysis, 16,906 genes (96.2%) were annotated with Gene Ontology (GO) terms (Additional file 4: Fig. S4), 1990 (11.3%) enzyme codes, 430 (2.5%) CAZyme domains, 10,487 (59.7%) PFAM domains, 1855 (10.5%) hypothetical proteins, and 1257 (7.1%) presented signal peptides (Additional file 8: Table S2). Nonetheless, to explore the role of *R. fluviale* LM-2 in lignin degradation at the genetic level, this work focused on aromatic degradation and lignin metabolism.

### Enzymes correlated with lignocellulose degradation and lignin metabolic pathways identified in the R. fluviale LM-2 genome

**Fig. 2:**
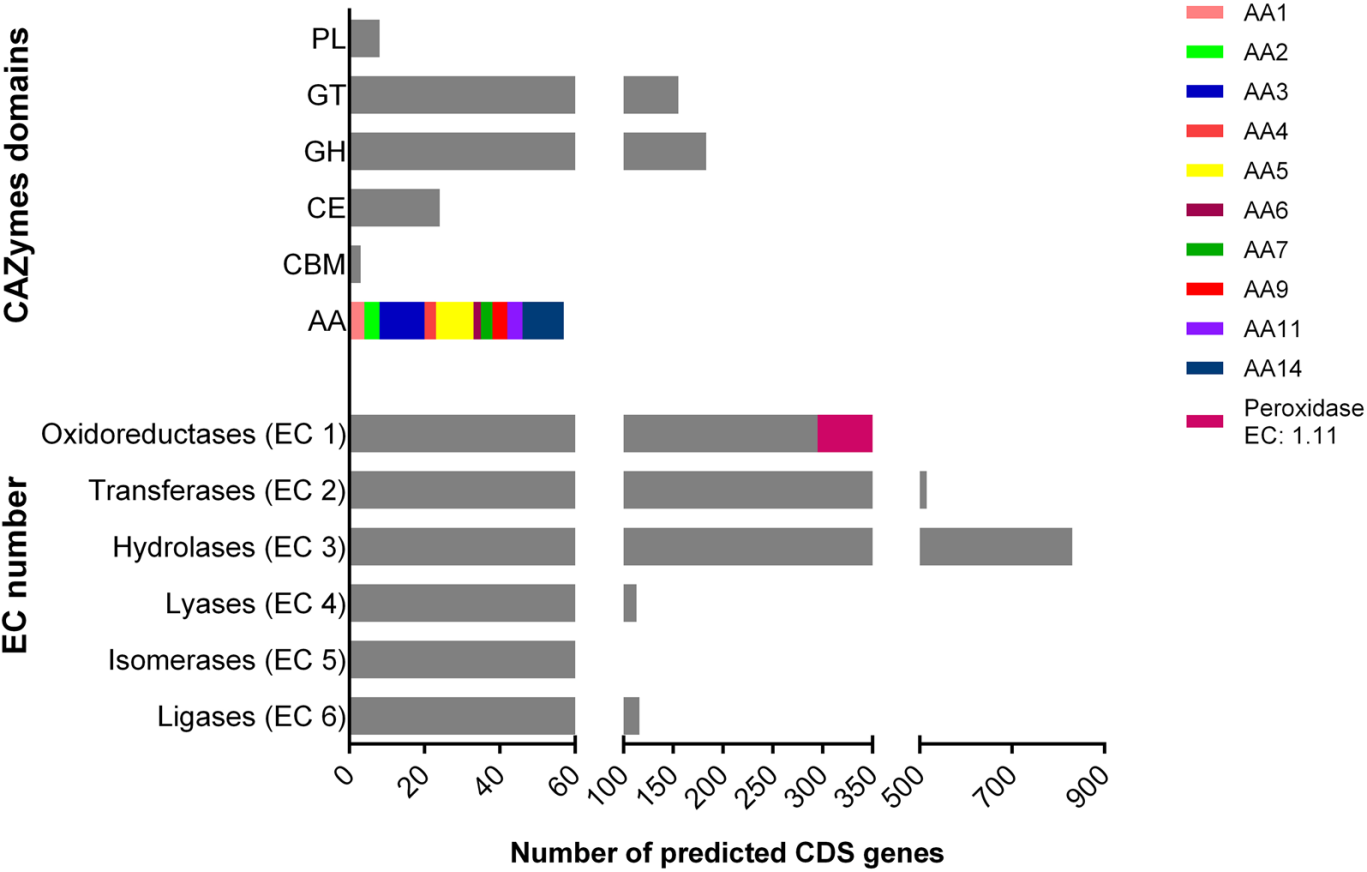
Enzyme classes and CAZy domains predicted in the *R. fluviale* LM-2 genome. Enzyme identification using Enzyme Commission number (EC number) and HMM-based dbCAN2 platform for CAZy domains (PL: polysaccharide lyases; GT: glycosyl transferases; GH: glycoside hydrolases; CE: carbohydrate esterases; CBM: carbohydrate-binding modules; AA: auxiliary activities)

Several enzymes involved in the first step of lignin degradation were identified in the *R. fluviale* LM-2 genome. Based on enzymatic functions [34], 61 genes were classified as peroxidases (EC 1.11.1) (Fig. 2), including four heme peroxidases (EC 1.11.1.14), nine catalases (EC 1.11.1.6), two glutathione peroxidases (EC 1.11.1.12), two Dyp-type peroxidases (EC 1.11.1.19), and CYP enzymes (EC 1.11.1.7). While class II heme peroxidases, Dyp-type peroxidases, and CYP are capable of degrading polymeric lignin [35, 36], catalases and glutathione peroxidases are responsible for controlling the levels of reactive oxygen species (ROS) generated during lignin oxidation, by regulating the intracellular levels of H_2_O_2_, and thus protecting the cells under stress conditions [37].

*R. fluviale* LM-2 genome contains several CAZymes, including 183 predicted glycoside hydrolases (GHs), 155 glycosyltransferases (GTs), eight polysaccharide lyases (PLs), and 24 carbohydrate esterases (CEs) (Fig. 2). Among the CAZymes involved in lignin degradation, 57 belonged to 10 distinct auxiliary activity (AA) families. For instance, four genes previously identified as those of heme peroxidases (EC 1.11.1.14) belong to the AA2 family, 12 genes coding for aryl alcohol oxidases (AAO) belong to the AA3 family, and 10 genes coding for GLOX belong to the AA5_1 subfamily. Moreover, four AAO and nine GLOX genes were predicted to code for signal peptides, indicating that the encoded proteins may be extracellularly localized and participate in lignin degradation.

**Fig. 3:**
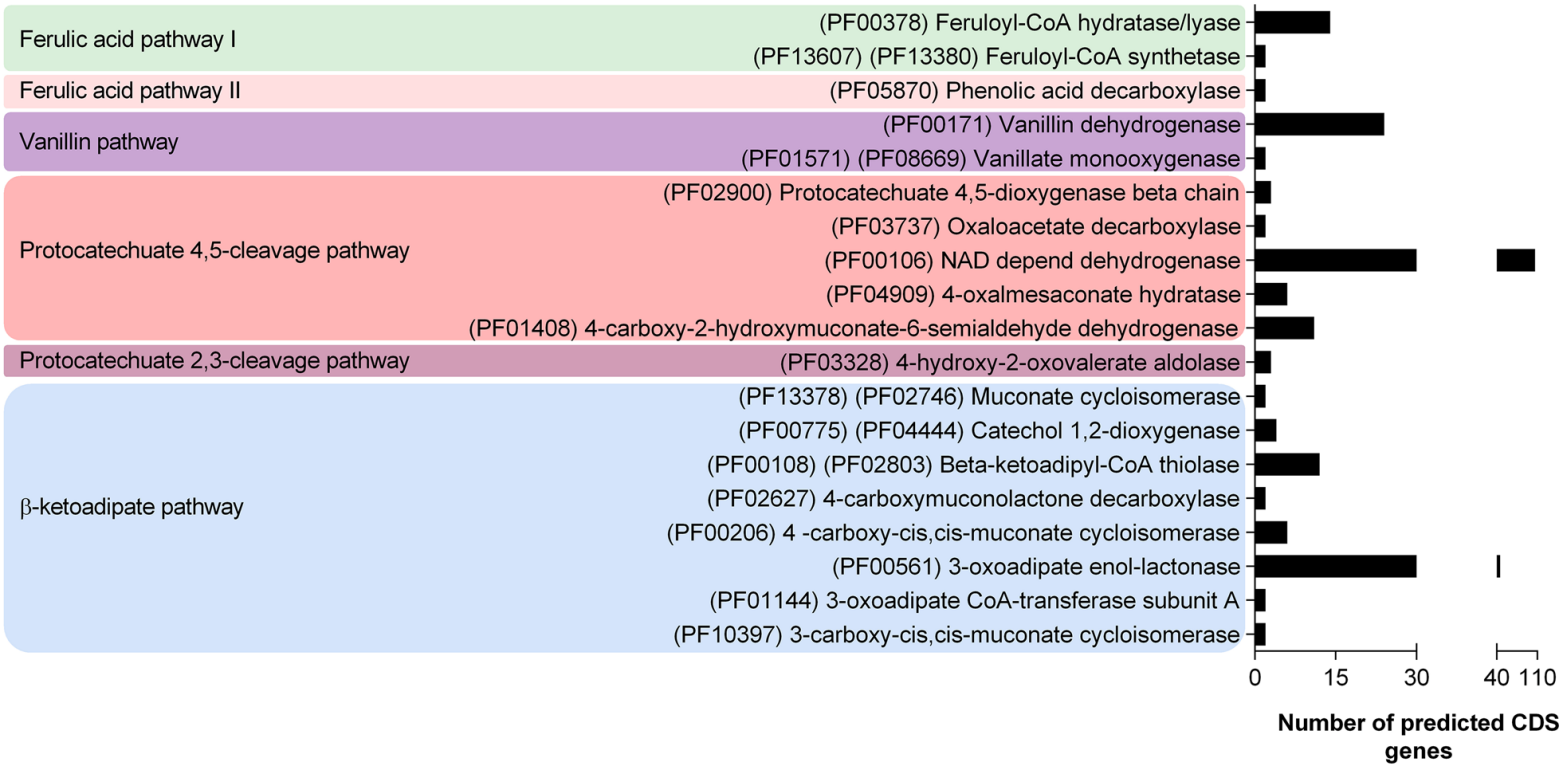
Predicted proteins encoding Pfam domains for aromatic compound degradation. The genes were identified in the *R. fluviale* LM-2 genome

In addition to oxidative enzymes, the intracellular pathways for lignin and aromatic catabolism were also investigated, reconstructing metabolic pathways based on Pfam domain prediction. Among the 17,565 proteins predicted, 31 β-etherase encoding sequences (Pfam numbers: PF02798, PF00043, and PF13417) participated in intracellular lignin degradation, and 246 encoded proteins were related to aromatic catabolism (Fig. 3). Among the predicted aromatic catabolic enzymes, two FCSs (PF13607 and PF13380), 14 FCHLs (PF00378), and two PDCs (PF05870) were identified, suggesting that *R. fluviale* LM-2 is potentially capable of catabolizing ferulic acid by two distinct pathways, named here as ferulic acid pathways I (via vanillin) and II (via 4-vinyl guaiacol) (Additional file 5: Fig. S5). Moreover, the identification of enzymes involved in protocatechuate 4,5 and protocatechuate 2,3-cleavage pathways and the β-ketoadipate pathway indicated that protocatechuate produced from the vanillin pathway can be converted to central intermediates through these three different pathways (protocatechuate 4,5-cleavage, protocatechuate 2,3-cleavage and β-ketoadipate pathways).

### Gene expression and production of ligninolytic enzymes

Transcriptome and secretome analyses were combined to investigate the ability of *R. fluviale* LM-2 to degrade lignin and metabolize lignin-derived aromatic compounds. Transcriptomic data contained an average of 25 million and 17 million reads for replicates with kraft lignin-containing medium and glucose control, respectively. RNA-seq analysis identified 15,986 distinct transcripts, of which 3618 were differentially expressed during cultivation on lignin-containing medium (*p* ≤ 0.05), including 1657 upregulated (Log_2_ fold change ≥ 1) and 1961 downregulated (Log_2_ fold change ≤ 1) transcripts (Additional file 6: Fig. S6). The Log_2_ fold change ranged from 8.5 to − 9.1, and the top ten upregulated and downregulated genes by fold change are listed in Additional file 9: Table S3. The top ten upregulated genes consisted mainly of hypothetical proteins and dehydrin, which is a stress response protein (DHN family protein). On the other hand, the top ten downregulated genes included sugar transporters and proteins identified from the expansin family which include cell wall modification proteins [38].

**Fig. 4:**
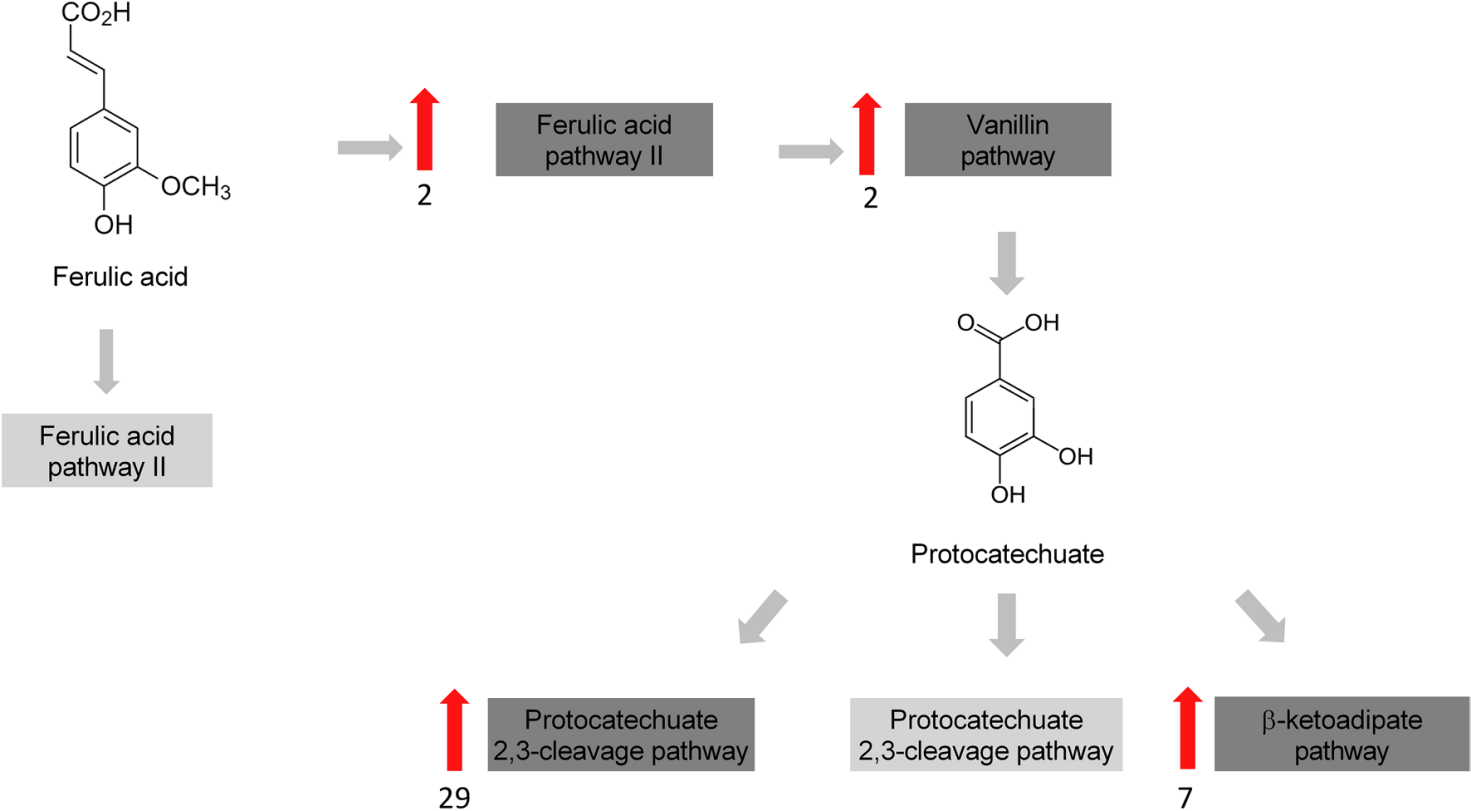
Differential expression of enzymes related to aromatic degradation identified in the *R. fluviale* LM-2 transcriptome. Red arrows show the number of upregulated genes identified by RNA sequencing. Upregulated genes: values of Log_2_ fold change > 1 and *p* value ≥ 0.05. No upregulated genes related to ferulic acid pathway II or the protocatechuate 2,3-cleavage pathway were identified under the conditions analyzed. Enzymes identified as upregulated: feruloyl-CoA hydratase/lyase, PF00378 (Ferulic acid pathway I); Vanillin dehydrogenase, PF00171 (Vanillin pathway); NAD-depend dehydrogenase, PF00106 (protocatechuate 4,5-cleavage pathway); 3-oxoadipate enol-lactonase, PF00561 and beta-ketoadipyl-CoA thiolase, PF00108/PF02803 (β-ketoadipate pathway)

Table 3 summarizes the upregulated genes encoding enzymes related to lignin depolymerization, including heme peroxidases (AA2), β-etherase, and CYP. No upregulated genes related to ferulic acid pathway II or protocatechuate 2,3-cleavage pathway were identified under the conditions analyzed. Therefore, under the analyzed conditions, the transcriptomic analyses indicated that *R. fluviale* LM-2 catabolizes ferulic acid preferentially through ferulic acid pathway I, followed by the vanillin, protocatechuate 4,5-cleavage, and β-ketoadipate pathways (Fig. 4). A finding that has indirect implications for lignin degradation, is that the stress response enzyme catalase (Table 3) and 45 genes of 229 major facilitator superfamily (MFS) transporters predicted in the *R. fluviale* LM-2 genome (Additional file 8: Table S2) were also upregulated during cultivation on lignin-containing medium.

**Table 3:** Expression profiles of ligninolytic genes in the *R. fluviale* LM-2 transcriptome

Gene ID	Log_2_ fold change	Padj	Description	Signal peptide
			*Stress response*	
RF_00400	4,487,506	0	Catalase	N
RF_00413	3,668,136	2.6E− 257	Catalase	N
RF_15827	2,058,997	0,005,316	Catalase	N
RF_03956	1,124,304	1.88E− 13	Catalase	N
			*Peroxidase/CAzyme domain*	
RF_03083	3,607,596	2.51E− 15	Cytochrome P450 (CYP)	N
RF_03010	2,439,883	5.95E− 08	Cytochrome P450 (CYP)	N
RF_07782	2,260,797	1.44E− 57	AA14	N
RF_07678	2,126,078	1.45E− 40	Cytochrome P450 (CYP)	N
RF_13258	2,067,324	2.23E− 69	Peroxidase (AA2)	N
RF_08081	1,873,513	0,026,431	Dye-decolorizing peroxidase	N
RF_09918	1,829,428	2.06E −56	Peroxidase (AA2)	N
RF_07773	1,605,494	8.62E− 35	AA14	Y
RF_03735	1,473,976	1.61E− 41	Cytochrome P450 (CYP)	N
RF_03638	1,472,094	0,010,065	Cytochrome P450 (CYP)	N
RF_10983	1,354,723	6.78E− 17	Cytochrome P450 (CYP)	N
RF_09992	1,202,432	2.25E− 13	AA14	Y
			*β-O-4 cleavage*	
RF_06343	1,688,851	6.45E− 55	β-Etherase	N
RF_17729	1,415,595	2.16E− 17	β-Etherase	N
RF_06332	1,377,958	1.02E− 32	β-Etherase	N

RNA sequence results were obtained after cell culture in kraft lignin-containing medium. Padj is the adjusted *p* value (≥ 0.05)

**Fig. 5:**
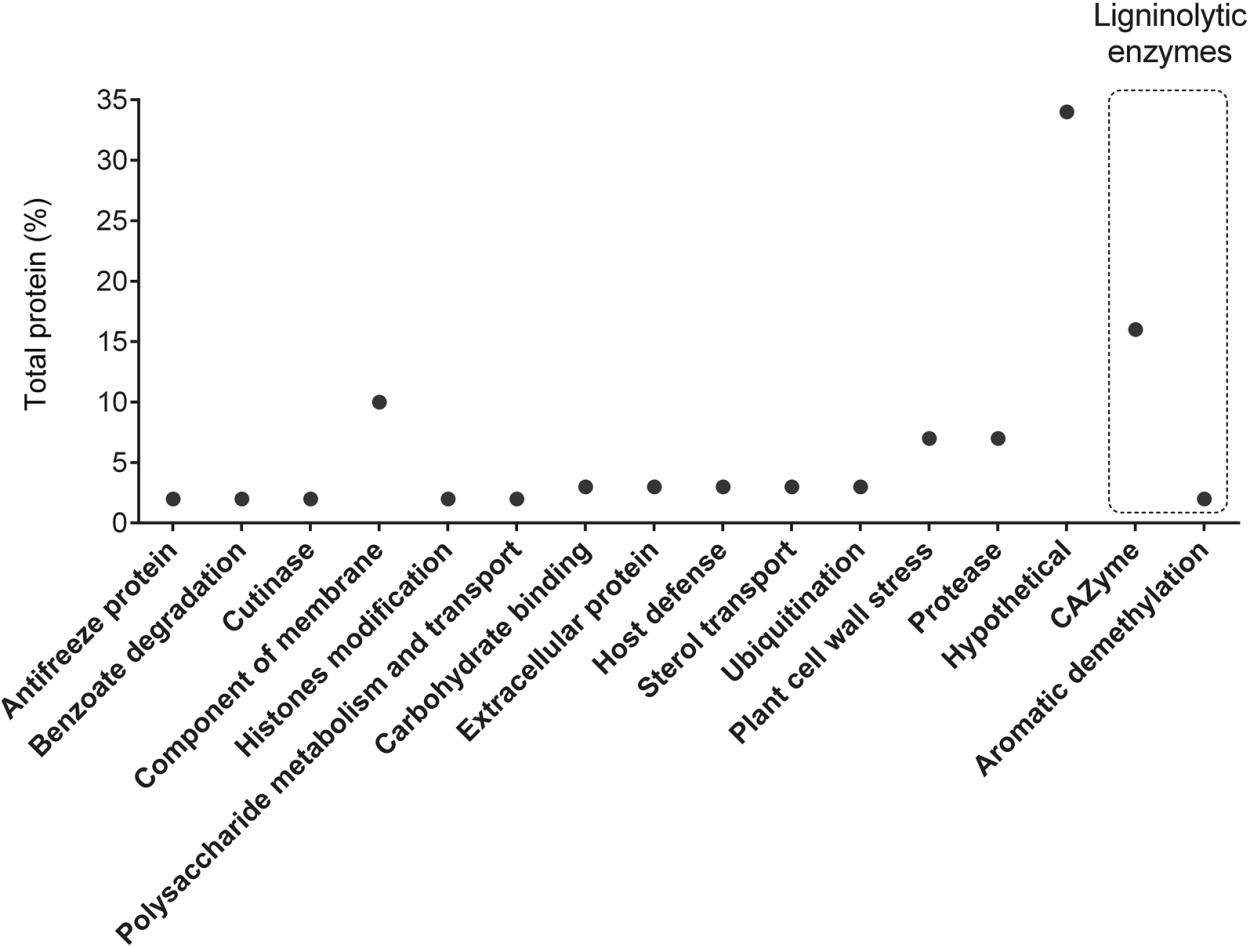
Functional categorization of proteins secreted by *R. fluviale* LM-2 identified through UHPLC–MS/MS. Cells were grown in minimal medium (YNB) supplemented with 1% kraft lignin for the assay. Two sequences for a GLOX (CAZyme-AA5_1) and one for CYP (aromatic demethylation) were also classified as ligninolytic enzymes

To identify secreted proteins related to lignin degradation, *R. fluviale* LM-2 cells were cultivated in lignin-containing medium. After 24 h, supernatant was collected for mass spectrometry and data processing. Sixty-one protein matches were identified (Table 4), with 271 unique peptides. Among these protein matches, 21 (34%) were identified as hypothetical proteins, 10 (16%) as CAZymes (coding for GT71 family, GH23 family, GH128 family, AA5_1 subfamily, and carbohydrate-binding molecules-CBM), 3 (5%) as ligninolytic enzymes (GLOX—AA5_1 and CYP) (Fig. 5), and 45 (74%) as signal peptides (Table 4). With regard to ligninolytic enzymes, two protein matches for the auxiliary activity enzyme GLOX – AA5_1 (with a signal peptide) and one protein match for CYP (without a signal peptide) were identified. Although *R. fluviale* LM-2 contains genes related to lignin-modifying peroxidases (AA2), none of these enzymes were detected in the secretome.

**Table 4:** *R. fluviale* LM-2 secreted proteins

ID Gene	Description	dbCAN2	Pfam	Signal peptide
RF_07791	3-Carboxymuconate cyclase	–	PF10282	N
RF_01894	Acid protease	–	PF00026	N
RF_01911	Acid protease	–	PF00026	N
RF_03151	Antifreeze glycopeptide AFGP poly	–	–	N
RF_15923	Aspartic peptidase A1	–	PF00026	Y
RF_04634	Aspartic peptidase A1	–	PF00026	Y
RF_08431	Barwin-related endoglucanase domain	–	–	Y
RF_07905	Carbohydrate-binding module family 13	CBM	–	Y
RF_07931	Carbohydrate-binding module family 13	CBM	–	Y
RF_01412	Copper radical oxidase	AA5_1	PF07250	Y
RF_10596	Copper radical oxidase	AA5_1	PF07250	Y
RF_08459	Cutinase	CE5	PF01083	Y
RF_08905	Cytochrome P450	–	PF00067	N
RF_08566	Expansin family	–	PF03330	Y
RF_08896	Expansin family	–	–	Y
RF_08894	Expansin family	–	–	N
RF_12837	Expansin family	–	PF03330	Y
RF_01475	Extracellular aldonolactonase	–	PF10282	N
RF_07820	Extracellular matrix FRAS1	–	–	Y
RF_05280	Glycoside hydrolase family 128	GH128	PF11790	Y
RF_05281	Glycoside hydrolase family 128	GH128	PF11790	Y
RF_05819	Glycoside hydrolase family 128	GH128	PF11790	Y
RF_05820	Glycoside hydrolase family 128	GH128	PF11790	Y
RF_14772	Glycoside hydrolase family 23	GH23	PF01464	Y
RF_03110	Glycosyltransferase family 71	GT71	PF01764	Y
RF_15534	Histone deacetylase HOS2	–	PF00850	N
RF_04368	Hypothetical protein	–	–	Y
RF_13136	Hypothetical protein	–	–	Y
RF_09132	Hypothetical protein	–	–	Y
RF_09130	Hypothetical protein	–	–	Y
RF_02059	Hypothetical protein	–	–	Y
RF_08909	Hypothetical protein	–	–	Y
RF_09346	Hypothetical protein	–	–	Y
RF_02025	Hypothetical protein	–	–	Y
RF_01570	Hypothetical protein	–	–	Y
RF_16610	Hypothetical protein	–	–	Y
RF_14805	Hypothetical protein	–	–	Y
RF_15339	Hypothetical protein	–	–	Y
RF_15335	Hypothetical protein	–	–	N
RF_10235	Hypothetical protein	–	–	N
RF_17543	Hypothetical protein	–	–	N
RF_05010	Hypothetical protein	–	–	N
RF_12461	Hypothetical protein	–	–	Y
RF_05776	Hypothetical protein	–	–	Y
RF_06525	Hypothetical protein	–	–	Y
RF_17040	Hypothetical protein	–	–	Y
RF_03290	Hypothetical protein	–	–	Y
RF_01252	Macrophage activating glycoprotein	–	–	N
RF_01247	Macrophage activating glycoprotein	–	–	Y
RF_01258	Macrophage activating glycoprotein	–	–	Y
RF_02177	Macrophage activating glycoprotein	–	–	Y
RF_13901	Macrophage activating glycoprotein	–	–	Y
RF_16668	Phosphatidylglycerol phosphatidylinositol transfer	–	PF02221	Y
RF_02006	Phosphatidylglycerol phosphatidylinositol transfer	–	PF02221	Y
RF_09948	Polyubiquitin	–	PF00240	N
RF_04571	Putative membrane protein	–	PF07767	N
RF_00438	Thaumatin-like protein	–	PF00314	Y
RF_17737	Thaumatin-like protein	–	PF00314	Y
RF_04689	Ubiquitin	–	PF00240	N
RF_13130	WSC domain	–	PF01822	Y
RF_04359	WSC domain	–	PF01822	Y

Secretome analysis after cultivation in medium containing 0.1% kraft lignin. **T**he protein sequences were analyzed on Blast2Go [68], for the predicted function description; on dbCAN2 [73], for CAZy domain identification; on Pfam [71]; and on SignalP v4 (72), for signal peptide identification

Collectively, gene expression and production of ligninolytic enzymes analysis confirmed that *R. fluviale* LM-2 can carry out the first and second steps of the lignin degradation, producing H_2_O_2_, which is the substrate of peroxidases and several enzymes involved in the conversion of phenolic compounds into central metabolic intermediates.

### Bioconversion of ferulic acid into 4-VG

Ferulic acid is the major hydroxycinnamic acid recovered from plant biomass and has a broad spectrum of antibacterial, anti-inflammatory, and antioxidant activities [39]. In addition, this phenolic compound is an important precursor for the production of high value-added chemicals such as vanillin and 4-VG [40], which are aromatic compounds used to impart vanilla flavor and clove aroma to food products, respectively.

**Fig. 6:**
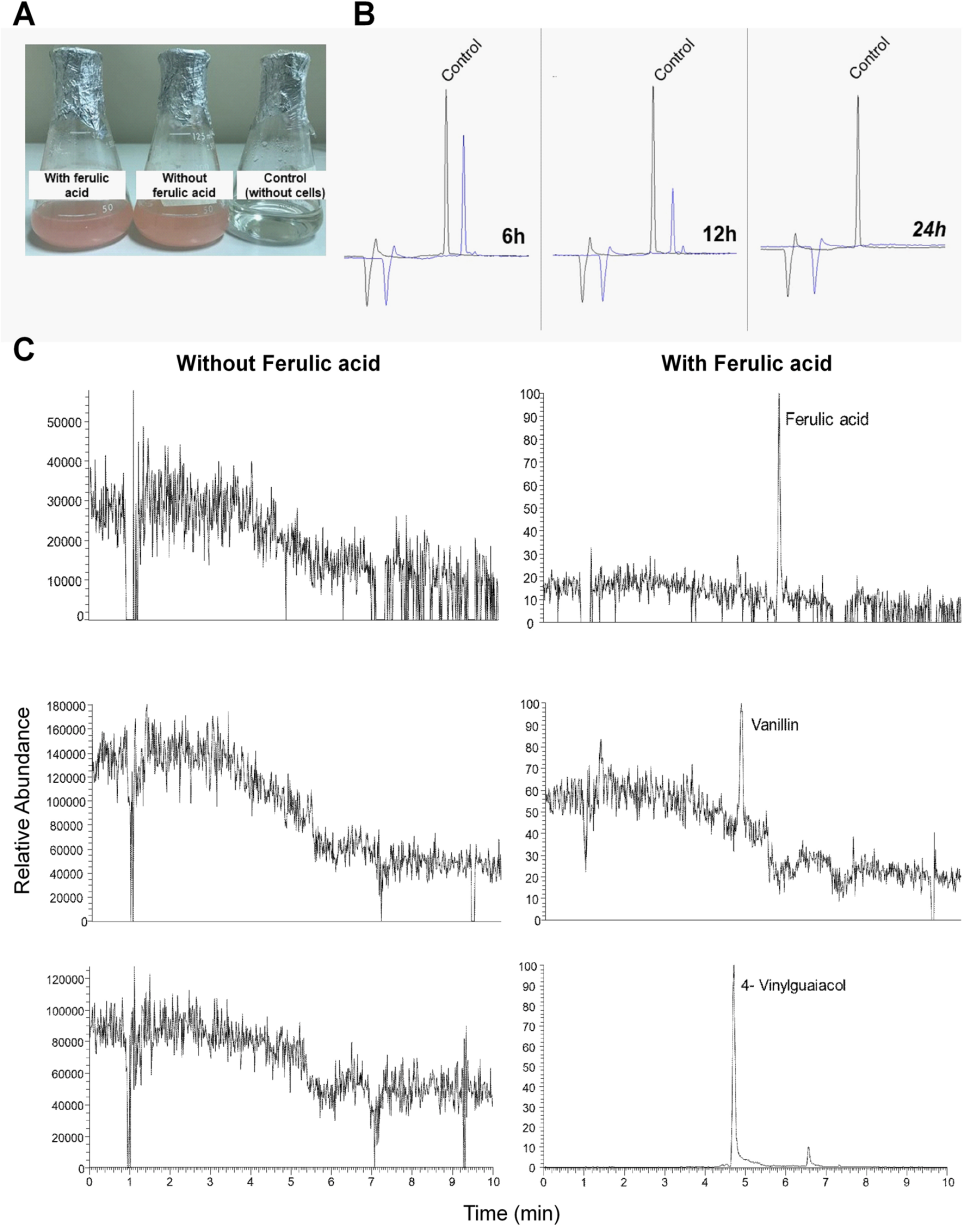
Evaluation of ferulic acid assimilation and catabolism by *R. fluviale* LM-2. **A** Cell culture in minimal medium (YNB) containing 0.1% glucose with/without 1.25 mM ferulic acid. Control: minimal medium (YNB) + 0.1% glucose + 1.25 mM ferulic acid without cells; **B** detection of ferulic acid by capillary electrophoresis (UV-214 nm) at different time points (6 h, 12 h and 24 h). Comparison between the culture containing ferulic acid and *R. fluviale* LM-2 cells and the control (without cells). **C** Intracellular metabolite identification after 12 h of cultivation. Comparison between the culture of *R. fluviale* LM-2 cells with/without ferulic acid in the medium. (Ferulic acid: MW 194 g/mol; C_10_H_10_O_4_; retention time 5.6 min), (4-vinyl-guaiacol: MW 150 g/mol; C_9_H_10_O_2_; retention time 4.7 min and 6.5) and (vanillin: MW 152 g/mol; C_8_H_8_O_3_; retention time 4.7 min)

To evaluate the ability of *R. fluviale* LM-2 to convert ferulic acid into 4-VG and vanillin, *R. fluviale* LM-2 cells were cultivated in minimal medium with and without ferulic acid. Capillary electrophoresis of the extracellular fluid indicated that ferulic acid was not degraded spontaneously during cultivation (negative control: minimal medium with ferulic acid), and *R. fluviale* LM-2 completely consumed this compound within 24 h (Fig. 6B). Secondly, ferulic acid and its possible conversion products (4-VG and vanillin) were detected intracellularly by mass spectrometry after 12 h of cultivation (Fig. 6C and Additional file 10: Table S4). For the latest analysis, the results of *R. fluviale* LM-2 cultivated with ferulic acid were compared with the results of *R. fluviale* LM-2 cultivated without ferulic acid.

Therefore, mass spectrometry analysis validated the presence of the two metabolic pathways for ferulic acid conversion predicted from the genomic analysis (Additional file 5: Fig. S5): one based on the sequential action of FCS and FCHL to produce vanillin (ferulic acid pathway I), and the other based on the decarboxylation of ferulic acid to produce 4-VG (ferulic acid pathway II), catalyzed by PDC.

## Discussion

To our knowledge, the genomic analysis of *R. fluviale* LM-2 in this study is the first one for this species, and as with other species from this genus, *R. fluviale* is capable of accumulating lipids (up to 50–70% of their dry weight) [41] and of tolerating inhibitory compounds [42]. For example, *R. fluviale* DMKU-RK253, which was isolated after enrichment of sugarcane leaf samples [41], accumulated high lipid levels after cultivation in glycerol containing medium [31]. Comparative genomic analysis revealed that *R. fluviale* LM-2 harbors a relatively large genome (50 Mb), with a larger set of predicted protein-coding genes (17,565) than *R. toruloides* (20.2 Mb, containing 8171 protein-coding genes) [43]. Genome size variation is a typical adaptive response in fungi to adapt to a specific habitat or ecological niche and involves genome duplication and translocation [44]. For instance, the genome of *Phanerochaete carnosa* shows a tandem duplication of ligninolytic genes compared to *P. chrysosporium* [45]. Furthermore, duplication of these genes could confer competitive advantage to ligninolytic organisms in nature, compared to other organisms that have less tolerance to the toxicity of aromatic compounds [46]. The large size of *R. fluviale* LM-2 genome was also observed based on de novo transcriptome assembly using Trinity, which was about 44 Mb with a completeness of 80%.

Multi-omic analysis of *R. fluviale* LM-2 elucidated a repertoire of genes, transcripts, and secreted proteins involved in lignin degradation, as well as the ability to convert lignin-derived aromatics into vanillin and 4-VG. *R. fluviale* LM-2 expresses heme peroxidases (AA2), β-etherases, and CYPs for lignin depolymerization, as well as several enzymes with a Pfam domain related to aromatic degradation. In contrast to the *Rhodotorula sp.* R2 [23], which secretes enzymes that act directly on lignin macromolecules, the AA2 enzyme from *R. fluviale* LM-2 was not secreted under the conditions analyzed. However, *R. fluviale* LM-2 appears to perform the first step of lignin degradation by secreting GLOX—AA5_1. Although aromatic compound metabolism by yeast is an uncommon phenotype, for example, *S. cerevisiae* INVSc1 Invitrogen^™^ uptakes low levels of mono-aryl compounds for further metabolism [47], oleaginous yeasts such as *R. fluviale* LM-2 have been extensively studied in this context since the products of aromatic metabolism (acetyl-CoA and pyruvate) are fatty acid biosynthesis precursors. For instance, Yaguchi and collaborators (2020) screened 36 yeast strains cultivated with several aromatic compounds, in which each species presented a unique metabolism and tolerance profile to these compounds [48]. Additionally, it is important to mention that the work described here focused on the secreted enzymes for lignin depolymerization. However, the whole proteomic analysis would also be helpful to improve the coverage of lignin-inducible genes in this yeast.

Collectively, the data indicated that during lignin catabolism, genes involved in stress response were upregulated in *R. fluviale* LM-2, probably in response to the toxicity of aromatic compounds. For example, enzymes responsible for controlling intracellular ROS production, such as catalases, were overexpressed in the presence of lignin. In addition, DNH was also overexpressed under the conditions analyzed. DHN has been characterized as a stress enzyme in plants, and is involved in membrane protection, cryoprotection of enzymes, and protection from reactive oxygen species [49, 50]. Beyond its demethylation role, CYP is also an essential component of the stress response system [51] and could therefore play a role in the adaptation of *R. fluviale* LM-2 to support ligninolytic pathways.

Among the transporters, MSF was upregulated in response to growth in lignin-containing medium, indicating its importance in lignin catabolism in *R. fluviale* LM-2. Members of this superfamily transport various small compounds, including aromatic compounds, across biological membranes [52]. Moreover, the overexpression of this transporter has been described as crucial for increasing protocatechuate conversion in *Sphingobium sp.* strain SYK-6 [53].

The biocatalytic conversion of ferulic acid can be useful for the production of desired chemicals [46, 54, 55]. Ferulic acid is usually converted to vanillin by FCS and FCHL with ATP consumption in two steps [56, 57]: (i) CoA-thioesterification of ferulic acid by FCS, and (ii) hydration of feruloyl-CoA by FCHL. The alternative catabolic route of ferulic acid through the 4-VG pathway is a detoxification process involving non-oxidative decarboxylation driven by the cofactor-free enzyme PDC [20]. Sequences coding a FCS, a FCHL, and a PDC were identified in the *R. fluviale* LM-2 genome based on Pfam domain analyses. This finding and the detection of vanillin and 4-VG after cultivation with ferulic acid indicated that these two ferulic acid catabolic pathways are functional in *R. fluviale* LM-2. Furthermore, similar to *R.* *toruloides* IFO0880 [24], *R. fluviale* LM-2 consumed all the ferulic acid added to the culture media before 24 h of growth.

## Conclusion

The present study shows that *R. fluviale* LM-2 possesses a wide spectrum of enzymes involved in lignin and phenylpropanoid degradation, which can be useful for lignin valorization strategies. Therefore, these results suggest that *R. fluviale* LM-2 could not only be classified as a ligninolytic yeast, but also as a degrader of polycyclic aromatic hydrocarbons and heterocyclic aromatic pollutants. In summary, the omics-based characterization of *R. fluviale* LM-2 opens new opportunities for biotechnological applications of this yeast. The availability of genomic data can support the genetic manipulation of this yeast and the development of lignin valorization strategies. In addition, this work uncovered functional ligninolytic pathways and novel genes, including FCS, FCHL, and PDC enzymes, that can be exported to other systems, such as model organisms, in biotechnology for the production of biofuels and bioproducts.

## Methods

### Isolation and identification

The yeast strain was identified in a lignin-degrading microbial consortium established on acidified black liquor generated from delignification of steam-exploded sugarcane bagasse (LW lignin) [30]. The yeast strain was isolated on separated agar plates containing: 1:1 (v/v) LW lignin; 0.25% (w/v) HW lignin and 0.1% (w/v) glucose (HW + G) [30]. The strain’s tolerance to different concentrations of kraft lignin was analyzed using a spot plating assay, in which several dilutions (10^−1^–10^−7^) of the cells were plated on agar supplemented with kraft lignin at four concentrations of 1%, 0.5%, 0.25%, and 0.125%.

For species identification, total DNA was extracted using the Fast DNA^®^ Spin Kit for Soil (MP-Biomedicals, Irvine, CA, USA), according to the manufacturer’s instructions. The quality and concentration of the extracted DNA were evaluated using 1.0% (m/v) agarose gel electrophoresis and by measuring the absorbance at 260 nm using a NanoDrop^®^ 2000c spectrophotometer (Thermo Fisher Scientific, Waltham, MA, USA). The sequences of the hypervariable internal transcribed spacer ITS1 and ITS2 regions were amplified by PCR using the following primers: ITS3 forward (GCATCGATGAAGAACGCAGC) and ITS4 reverse (TCCTCCGCTTATTGATATGC). The resulting sequences were compared with those in the GenBank nonredundant nucleotide sequence database using the BLASTn algorithm [58].

### Genome sequencing and assembly

For genome sequencing, paired-end and mated-pair libraries were constructed using the Nextera XT DNA Sample Prep Kit and Nextera® Mate Pair Sample Preparation Kit (Illumina, San Diego, CA, USA), respectively. Libraries were quantified and quality checked using the KAPA library quantification kit (Merck, Darmstadt, Germany) and Bioanalyzer high-sensitivity DNA chips (Agilent, Santa Clara, CA, USA), and then sequenced on an Illumina MiSeq Platform using 2 × 300 bp, according to the manufacturer’s instructions.

Illumina reads of different sizes were first filtered to remove adapters and low-quality reads using NextClip [59] and Trimmomatic 0.32 [60] using default settings. The genome was de novo assembled using Velvet 1.2.10 [61] and SSPACE [62] was used for scaffolding using the mated-pair reads. Pilon [63] was used to further improve genome assembly. Gene calling was performed using the Maker pipeline [64] using Augustus [65] and SNAP [66]. Genome completeness was assessed through BUSCO v2 [67].

Functional annotation was performed using Blast2GO [68], InterProScan [69], Swiss-Prot [70] and Pfam [71] to predict motifs, domains, and other signatures. Signal peptides were predicted using SignalP, version 4 [72]. Comprehensive analysis of CAZymes was performed using the HMM-based dbCAN2 platform using HMMER and dbCAN (*E*-value < 1e− 15 and coverage > 0.35) [73].

### Growth conditions and transcriptome analysis

After overnight cultivation in YPD liquid medium (1% yeast extract, 2% peptone, and 2% glucose), yeast cells were harvested and washed three times with phosphate-buffered saline (PBS; 137 mM NaCl, 2.7 mM KCl, 10 mM Na_2_HPO4, and 2 mM KH_2_PO_4_). The cells were inoculated to a final OD_600_ of 0.2 and cultivated in triplicate for 16 h in Bushnell Haas Broth (Sigma–Aldrich, San Luis, MO, USA), pH 7.0, supplemented with 0.1% kraft lignin (Sigma–Aldrich, 471,003) and 0.1% glucose, or 0.1% glucose as the sole carbon source. 3,5-dinitrosalicylic acid (DNS) was used to estimate reducing sugars (data not shown).

Although *R. fluviale* LM-2 has a high tolerance to different concentrations of kraft lignin and can metabolize phenolic compounds, as described in the manuscript, the yeast could not grow well in liquid media with lignin as the only carbon source (data not shown). Thus, to proceed with the transcriptome analysis, it was added to the culture media 0,1% glucose consumed in less the 4 h by the yeast (data not shown).

Cells were harvested by centrifugation at 4000 rpm for 10 min, and total RNA was preserved using TRIzol^®^ (Thermo Fisher Scientific, Waltham, MA, USA), followed by extraction and purification using the RNeasy Plant MiniKit (QIAGEN, Hilden, Germany). The quantity and quality of the extracted RNA were determined using a NanoDrop^®^ 2000c spectrophotometer (Thermo Fisher Scientific, Waltham, MA, USA) and a 2100 Bioanalyzer platform (Agilent, Santa Clara, CA, USA), with a minimum RIN (RNA integrity number) of 7.0 [74].

Libraries were prepared using a TruSeq^®^ Stranded Total RNA Library Prep Kit (Illumina, San Diego, CA, USA). Quality and quantity were determined using capillary electrophoresis on a 2100 Bioanalyzer platform (Agilent, Santa Clara, CA, USA) and the KAPA Library Quantification Kit for Illumina (Merck, Darmstadt, Germany), respectively. The libraries were sequenced using the Illumina HiSeq 2500 platform (Illumina, San Diego, CA, USA), according to the manufacturer’s instructions.

Reads were preprocessed as described previously for the genome libraries, and evaluation and filtration of the rRNA were performed using SortmeRNA. The filtered data were mapped against the *R. fluviale* LM-2 reference genome sequenced in this study using the Tophat2 algorithm [75]. Differential gene expression analysis was based on counting data and was performed with R using the Bioconductor DESeq2 package [76] through paired comparisons against the control (medium lacking lignin). Transcripts showing differential expression (log2-fold change ≥ 1 and ≤ -1) relative to the control were determined using *p* ≤ 0.05 as the threshold. A volcano plot was generated using R scripts with the log2-fold change value as the input.

### Growth conditions for secretome analysis

After overnight preculture in YPD broth (1% yeast extract, 2% peptone, and 2% glucose), the cells were harvested by centrifugation (4,000 rpm, 10 min), washed with 30 ml of PBS, and inoculated into a flask containing 100 ml yeast nitrogen base (YNB) with amino acids (Sigma-Aldrich—Y1250) supplemented with 0.1% kraft lignin and 0.1% glucose to an initial OD_600_ of 0.1. After 24 h, the cultures were centrifuged (4000 rpm, 10 min), and the supernatants were filtered through 0.45 μm and 0.2 μm MF-Millipore^®^ membrane filters (Merck, Darmstadt, Germany) to remove residual cells. Protein content was concentrated using Vivaspin 20 ultrafiltration spin columns (Sartorius Stedim, Gottingen, Germany) with a molecular mass cutoff of 3 kDa, and quantified using the Bradford assay (BioRad^®^, Hercules, CA, USA) [77]. The proteins were separated by 10% SDS–PAGE and the protein bands were excised and analyzed by mass spectrometry.

### Secretome mass spectrometry analysis and data processing

For secretome analysis, aliquots of 25 µg of the concentrated supernatant were subjected to SDS–PAGE in triplicates, and the protein bands were excised and analyzed using Micro LC–MS/MS QTof XEVO G2 XS equipment (Waters, Milford, MA, USA) at the Life Sciences Core Facility (LaCTAD, UNICAMP, Campinas, SP, Brazil). The columns were equilibrated with 93% mobile phase A (0.1% formic acid in water) and 7% mobile phase B (0.1% formic acid in acetonitrile) at 40 ℃. Peptides were separated from the C18 Trap column (Waters, Milford, MA, USA) by gradient elution (7% to 40% acetonitrile) on an ACQUITY UPLC M-Class HSS T3 analytical column (Waters, Milford, MA, USA).

Data-independent acquisition (MSE) was carried out by operating the instrument in positive ion V mode, applying the MS and MS/MS functions over 0.5 s intervals with 6 V low energy and 15–45 V high energy collision, to obtain the peptide mass to charge ratio (*m/z*) and product ion information, for deducing the amino acid sequence. The capillary voltage and source temperature were set to 3.0 kV and 80 ℃, respectively. To correct the mass drift, the internal mass calibrant leucine enkephalin (556.2771 Da) was infused every 30 s through a lock spray ion source at a flow rate of 3 µL/min. Peptide signal data were collected between 100 and 2000 m*/z* values.

Proteins present in the samples were identified through comparison with the protein sequences previously predicted in the genome analysis, and by setting the minimum number of fragment ion matches per peptide and protein to three and five, respectively. The false positive discovery rate (FDR) was set at 4%. The FDR for peptide and protein identification was determined based on the search of a reversed database, which was generated automatically using ProteinLynx Global SERVER™ (PLGS) software (Waters, Milford, MA, USA), by reversing the sequence of each entry. All protein hits were identified at a confidence level of  > 95%. Raw data processing and protein identification were performed using the ProteinLynx Global SERVER 3.0.3 (Waters, Milford, MA, USA).

### Yeast cultivation and secondary metabolite extraction

After overnight preculture in YPD broth, yeast was cultivated in minimal medium YNB with amino acids supplemented with 0.1% glucose with and without 1.25 mM ferulic acid (Sigma–Aldrich, 128,708) to an initial OD_600_ of 0.1. The medium without cells was used as a control for compound degradation. Cultures were sampled after 6, 12, 24, 48, 72, and 96 h for further capillary electrophoresis (for extracellular fluid) and mass spectrometry (for intracellular fluid).

For analysis of extracellular fluid by mass spectrometry, the cultures were centrifuged (8000 rpm, 10 min), and the supernatant was used for secondary metabolite extraction using ethyl acetate (1:1). The organic phase was collected and dried using a centrifugal vacuum concentrator (Speed Vac Vacuum Concentrators, Thermo Fisher Scientific, Waltham, MA, USA). For intracellular secondary metabolite evaluation, intracellular compounds were extracted with 1 mL of MeOH and formic acid solution (0.1% v/v) for 40 min in an ultrasonic bath. The extracts were centrifuged (10,000 rpm for 10 min), and the supernatants were collected and dried using a centrifugal vacuum concentrator (SpeedVac Vacuum Concentrators; Thermo Fisher Scientific, Waltham, MA, USA). Before mass spectrometry, both extracts were resuspended in 1 ml MeOH and filtered using a 0.22-μm pore-size filter with a hydrophobic polytetrafluoroethylene (PTFE) membrane. All extractions were performed in triplicates.

### UHPLC–MS/MS analysis

Liquid chromatography analysis was performed using an UltiMate 3000 UHPLC coupled to a high-resolution Orbitrap Q-Exactive mass spectrometer (Thermo Fisher Scientific, Waltham, MA, USA) with an electrospray ionization source (RESI-II) set to 3515 V. Chromatography was performed on an Accucore C_18_ column (2.6 μm pore size, 2.1 mm × 100 mm) (ThermoFisher Scientific, Waltham, MA, USA). For gradient elution, 0.1% formic acid in water (solvent A) and 0.1% acetonitrile in water (solvent B) were used, and the eluent profile (A:B) was as follows:0 -10 min 5% solvent B, 10–15 min 5% to 98% solvent B, 15–16.2 min 98% to 5% solvent B and 16.2–25 min 5% solvent B. The flow rate was set at 0.2 ml min^−1^, and the injection volume was 3 μL. The voltage and temperature of the capillary were set to + 3.5 kV and at 250 ℃, respectively. The analyses were performed using collision energies of 20, 30, and 40 eV. The parameters of the MS analysis were set in positive ion mode ionization [M + H]^+^, with an *m/z* range of 115 to 1500, and the six most intense ions were selected for automatic fragmentation (Auto MS/MS). All operations and spectral analyses were performed using Xcalibur software, version 3.0.63 (ThermoFisher Scientific, Waltham, MA, USA).

### Sequence accession numbers

The draft genome sequence and transcriptome of *R. fluviale* LM-2 were deposited in the EMBL-EBI (European Molecular Biology Laboratory-European Bioinformatics Institute) database under the accession number PRJNA817419 (Genome: SRX14500032-35; Transcriptome: SRX14526891-SRX14526902).

## Supplementary Information

**Additional file 1: Figure S1.** Analysis of *R. fluviale *LM-2 tolerance to different concentrations of kraft lignin. *R. fluviale *LM-2 was pre-cultured in YPD medium for 24 h and several dilutions (10-1 to 10-7) were prepared for a spot plating assay. *R. fluviale *LM-2 was cultured in agar plates for 72 h with 1X YNB minimal medium containing kraft lignin in four concentrations: 1%, 0.5%, 0.25% and 0.125%. The positive control is an agar plate with 1X minimal medium.

**Additional file 2: Figure S2.** Phylogenetic trees based on the ITS regions of *R. fluviale *LM-2. Sequences of the closest relatives were obtained from a BLASTn search against the NCBI nonredundant database using the ITS sequences as queries. Alignments were constructed using MAFFT (79), and a phylogenetic tree was constructed using RAxML (80) with the GTR+Gamma model and bootstrap algorithm with an automatic option. The results tree was visualized and manually edited using iTOL (https://itol.embl.de). The full circles on the branches represent the percentages of bootstrap replications.

**Additional file 3: Figure S3.** Physiological test for the differentiation of *R. fluviale *from closely related species, *R. azoricum*. The cells were cultivated in liquid (**A **and **B**) and solid (**C**) media (YPD 2%) and incubated at 30 and 37 °C. Gadanho and collaborators (2001) reported that although *R. fluviale *and *R. azoricum *exhibit high similarity in the D1/D2 domain sequence (two mismatches), these species showed low reassociation values in DNA–DNA reassociation experiments, confirming that they are distinct species (81). In addition, Sampaio characterized physiological differences between the close species, in which *R. fluviale *could grow at 30 and 37 °C, while *R. azoricum *could not grow at 37 ℃ (32).

**Additional file 4: Figure S4.** Most abundant Gene ontology (GO) terms assigned to the *R. fluviale *LM-2 genome. Only the top ten GO terms for each category are represented. GO term categories: biological process (green); cellular component (pink); molecular function (blue). The x-axis indicates the number of genes assigned to the same GO term. One unigene may be matched to multiple GO terms.

**Additional file 5: Figure S5.** Outline of ferulic acid catabolism by *R. fluviale *LM-2. Based on genomic analysis, two catabolic pathways were predicted for ferulic acid bioconversion. A) Ferulic acid pathway I: ferulic acid conversion into vanillin in two steps with ATP consumption: i) CoA-thioesterification of ferulic acid by ferulic acid synthetase (FCS); ii) hydration of feruloyl-CoA by ferulic acid hydratase lyase (FCHL). B) Ferulic acid pathway II: cofactor-independent ferulic acid decarboxylation into 4-vinyl guaiacol (4-VG) by a phenolic acid decarboxylase (PDC).

**Additional file 6: Figure S6.** Differential gene expression analysis of *R. fluviale *LM-2 in response to kraft lignin. A) Volcano plot. X-axis: Log2 fold change (with lignin/without lignin). Y-axis: negative log10-adjusted *p* value. Red data points indicate upregulated transcripts, green data points indicate downregulated transcripts, and gray data points indicate nonmodulated genes. B) Number of up- and downregulated and nonmodulated genes.

**Additional file 7: Table S1.** Genomic reads details.

**Additional file 8: Table S2. **Description and gene expression profile of the ***R. fluviale ***LM-2 genome (Excel table). S2.1) List of *R. fluviale *LM-2 genes (IDs) with functional prediction protein, protein length, EC number and GO terms based on Blast2Go (69) analysis. The presence or absence of signal peptides was identified using SignalP (73). The Cazy domain was identified using dbCAN (74). For the Log2Fold change values, *p* ≤ 0.05 was considered statistically significant. Log2 fold change ≥ 1 was considered indicative of upregulation and Log2 fold change ≤ 1 was considered indicative of downregulation. S2.2) Number of genes with a GO separated by GO term categories: biological process, cellular component, and molecular function. S2.3) Pfam domain for each gene with the name of the enzyme for aromatic degradation and the aromatic pathway to which it is related. S2.4) Expression profiles of ligninolytic and aromatic compound degradation genes in the *R. fluviale *LM-2 transcriptome.

**Additional file 9: Table S3.** Top 10 up- and downregulated genes of *R. fluviale *LM-2.

**Additional file 10: Table S4.** Phenolic compounds detected by UHPLC–MS/MS analysis.

## Data Availability

All data generated or analyzed during this study are included in this published article and its supplementary information files. In addition, the draft genome sequence and transcriptome were deposited in the EMBL-EBI database under the accession number PRJNA817419 (Genome: SRX14500032-35; Transcriptome: SRX14526891-SRX14526902).

## Abbreviations

4-VG: 4-Vinylguaiacol
AA: Auxiliary activity
AAO: Aryl alcohol oxidases
BUSCO: Benchmarking Universal Single-Copy Orthologous
CE: Carbohydrate esterase
CYP: Cytochrome P450
DNS: 3,5-Dinitrosalicylic acid
EMBL-EBI: European Molecular Biology Laboratory-European Bioinformatics Institute
FCHL: Feruloyl-CoA hydratase-lyase
FCS: Feruloyl-CoA synthetase
FDR: False positive discovery rate
G: Guaiacyl
GH: Glycoside hydrolase
GLOX: Glyoxal oxidases
GO: Gene Ontology
GT: Glycosyltransferases
H: P-Hydroxyphenyl
HW: High-molecular-weight lignin
HW + G: High-molecular-weight lignin with glucose
LiP: Lignin peroxidase
LW: Low-molecular-weight lignin
MFS: Major facilitator superfamily transporters
MnP: Manganese peroxidase
MSE: Data-independent acquisition
PBS: Phosphate-buffered saline
PDC: Phenolic acid decarboxylase
PL: Polysaccharide lyases
PLGS: ProteinLynx Global SERVER™
PTFE: Polytetrafluoroethylene
RIN: RNA integrity number
ROS: Reactive oxygen species
S: Syringyl
UHPLC–MS/MS: Ultra-high-performance liquid chromatography coupled with mass spectrometry
YNB: Yeast nitrogen base
