# Supplementary material for: Integrative omics analyses of the ligninolytic Rhodosporidium fluviale LM-2 disclose catabolic pathways for biobased chemical production
Source: Biotechnol Biofuels Bioprod. 2023 Jan 9;16:5. doi: 10.1186/s13068-022-02251-6 (PMC9830802; doi:10.1186/s13068-022-02251-6)
Supplement: Supplementary file 1 — Additional file 1: Figure S1. Analysis of R. fluviale LM-2 tolerance to different concentrations of kraft lignin. R. fluviale LM-2 was pre-cultured in YPD medium for 24 h and several dilutions (10-1 to 10-7) were prepared for a spot plating assay. R. fluviale LM-2 was cultured in agar plates for 72 h with 1X YNB minimal medium containing kraft lignin in four concentrations: 1%, 0.5%, 0.25% and 0.125%. The positive control is an agar plate with 1X minimal medium. [file 13068_2022_2251_MOESM1_ESM.docx]

**
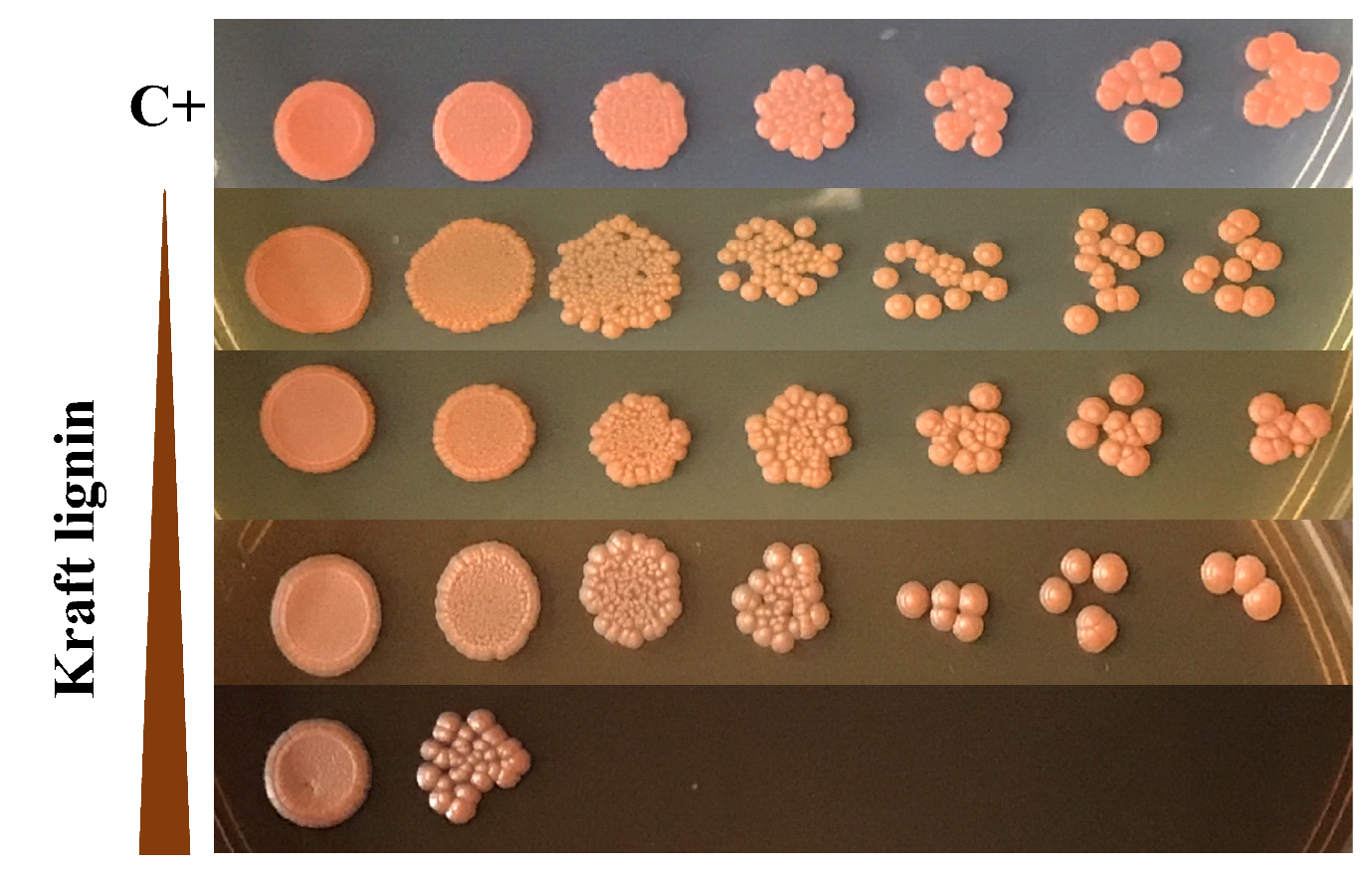
**

**Figure S1. Analysis of *R. fluviale* LM-2 tolerance to different concentrations of kraft lignin.** *R. fluviale* LM-2 was pre-cultured in YPD medium for 24 h and several dilutions (10^-1^ to 10^-7^) were prepared for a spot plating assay. *R. fluviale* LM-2 was cultured in agar plates for 72 h with 1X YNB minimal medium containing kraft lignin in four concentrations: 1%, 0.5%, 0.25% and 0.125%. The positive control is an agar plate with 1X minimal medium.
