# Supplementary material for: Integrative omics analyses of the ligninolytic Rhodosporidium fluviale LM-2 disclose catabolic pathways for biobased chemical production
Source: Biotechnol Biofuels Bioprod. 2023 Jan 9;16:5. doi: 10.1186/s13068-022-02251-6 (PMC9830802; doi:10.1186/s13068-022-02251-6)
Supplement: Supplementary file 2 — Additional file 2: Figure S2. Phylogenetic trees based on the ITS regions of R. fluviale LM-2. Sequences of the closest relatives were obtained from a BLASTn search against the NCBI nonredundant database using the ITS sequences as queries. Alignments were constructed using MAFFT (79), and a phylogenetic tree was constructed using RAxML (80) with the GTR+Gamma model and bootstrap algorithm with an automatic option. The results tree was visualized and manually edited using iTOL (https://itol.embl.de). The full circles on the branches represent the percentages of bootstrap replications. [file 13068_2022_2251_MOESM2_ESM.docx]

**
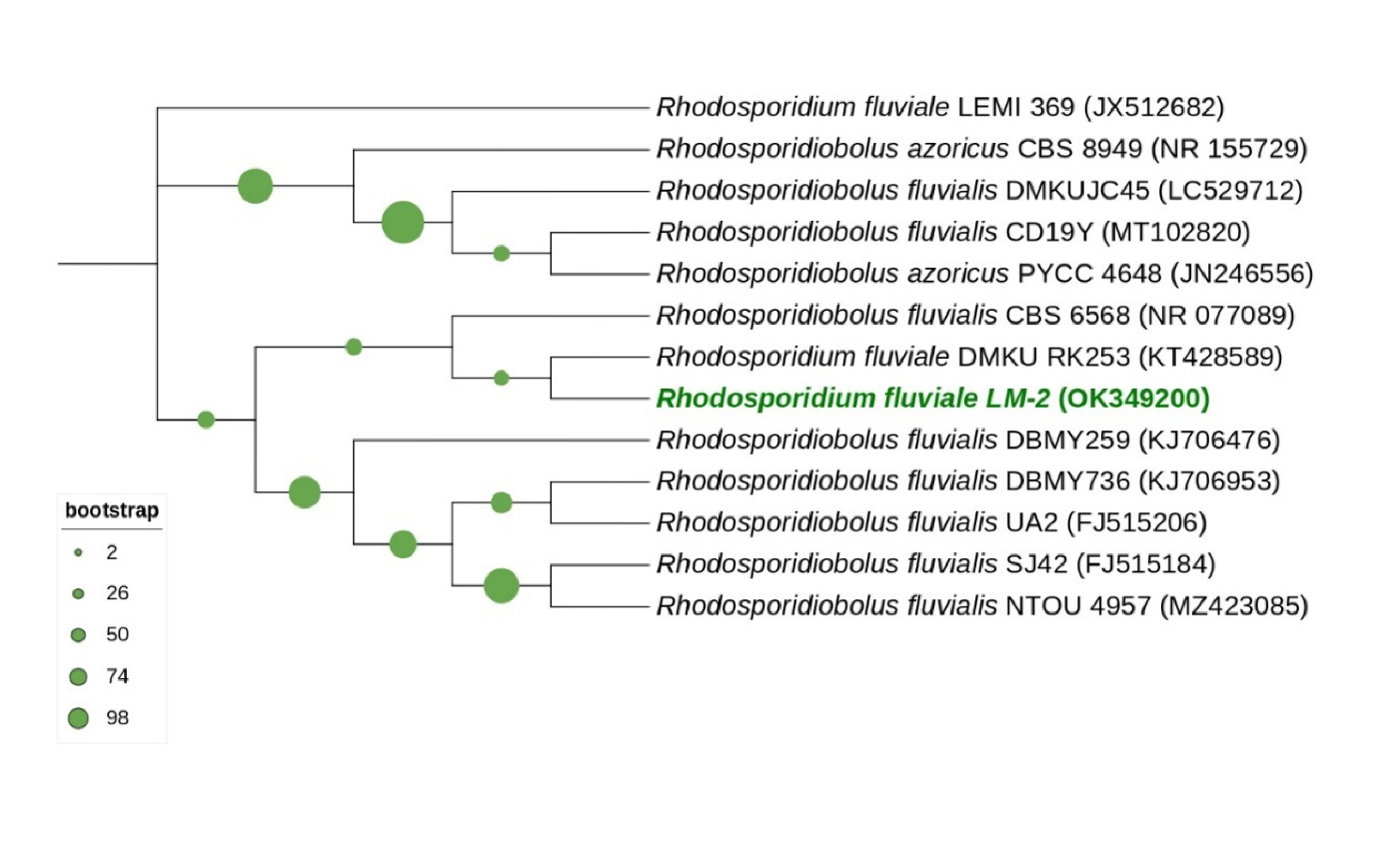
**

**Figure S2. Phylogenetic trees based on the ITS regions of *R. fluviale* LM-2.** Sequences of the closest relatives were obtained from a BLASTn search against the NCBI nonredundant database using the ITS sequences as queries. Alignments were constructed using MAFFT (79), and a phylogenetic tree was constructed using RAxML (80) with the GTR+Gamma model and bootstrap algorithm with an automatic option. The results tree was visualized and manually edited using iTOL (https://itol.embl.de). The full circles on the branches represent the percentages of bootstrap replications.
