# Supplementary material for: Integrative omics analyses of the ligninolytic Rhodosporidium fluviale LM-2 disclose catabolic pathways for biobased chemical production
Source: Biotechnol Biofuels Bioprod. 2023 Jan 9;16:5. doi: 10.1186/s13068-022-02251-6 (PMC9830802; doi:10.1186/s13068-022-02251-6)
Supplement: Supplementary file 3 — Additional file 3: Figure S3. Physiological test for the differentiation of R. fluviale from closely related species, R. azoricum. The cells were cultivated in liquid (A and B) and solid (C) media (YPD 2%) and incubated at 30 and 37 °C. Gadanho and collaborators (2001) reported that although R. fluviale and R. azoricum exhibit high similarity in the D1/D2 domain sequence (two mismatches), these species showed low reassociation values in DNA–DNA reassociation experiments, confirming that they are distinct species (81). In addition, Sampaio characterized physiological differences between the close species, in which R. fluviale could grow at 30 and 37 °C, while R. azoricum could not grow at 37 ℃ (32). [file 13068_2022_2251_MOESM3_ESM.docx]

**
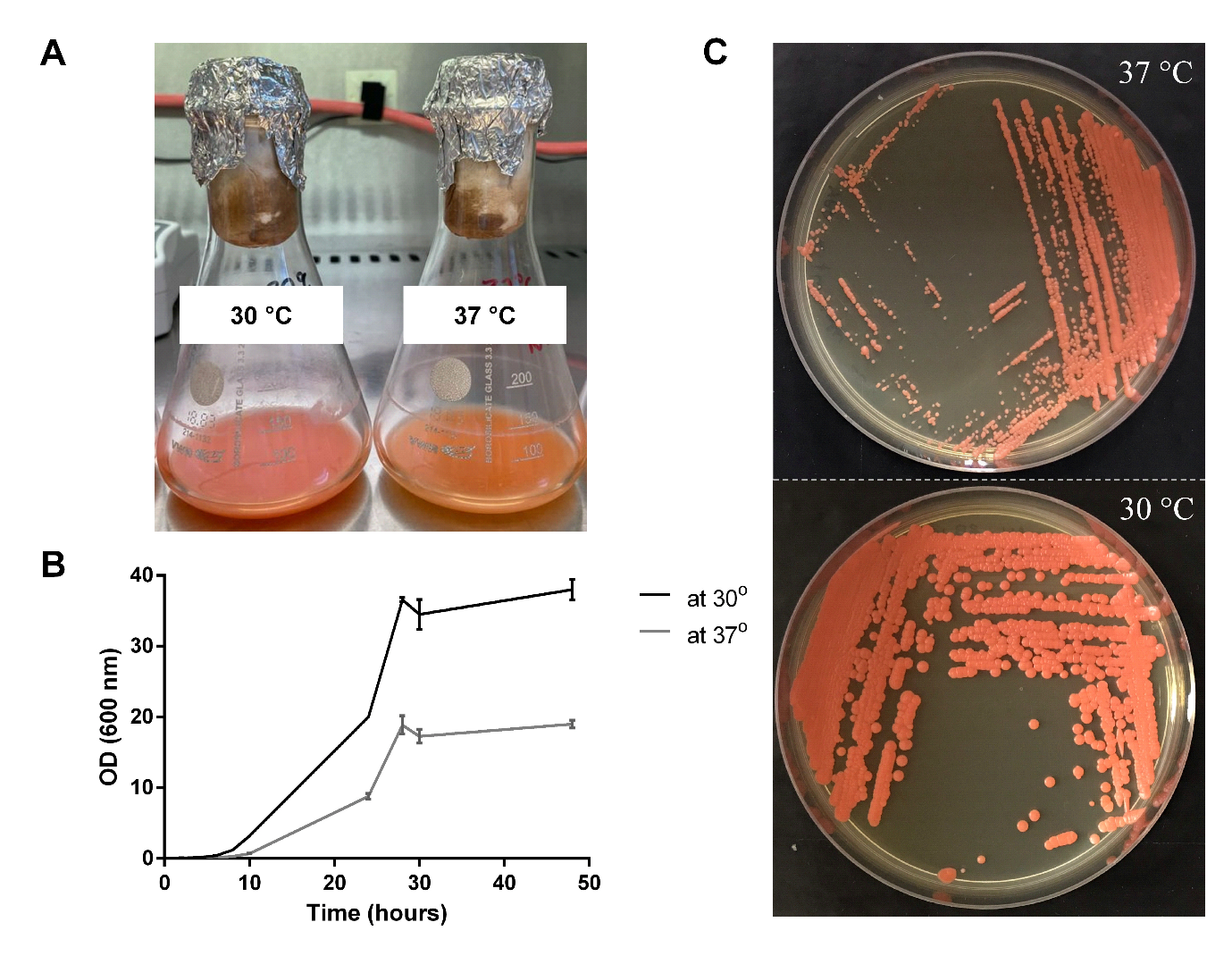
**

**Figure S3. Physiological test for the differentiation of *R. fluviale* from closely related species, *R. azoricum***. The cells were cultivated in liquid (**A** and **B**) and solid (**C**) media (YPD 2%) and incubated at 30 and 37 °C. Gadanho and collaborators (2001) reported that although *R. fluviale* and *R. azoricum* exhibit high similarity in the D1/D2 domain sequence (two mismatches), these species showed low reassociation values in DNA–DNA reassociation experiments, confirming that they are distinct species (81). In addition, Sampaio characterized physiological differences between the close species, in which *R. fluviale* could grow at 30 and 37 °C, while *R. azoricum* could not grow at 37 °C (32).
