# Supplementary material for: Integrative omics analyses of the ligninolytic Rhodosporidium fluviale LM-2 disclose catabolic pathways for biobased chemical production
Source: Biotechnol Biofuels Bioprod. 2023 Jan 9;16:5. doi: 10.1186/s13068-022-02251-6 (PMC9830802; doi:10.1186/s13068-022-02251-6)
Supplement: Supplementary file 4 — Additional file 4: Figure S4. Most abundant Gene ontology (GO) terms assigned to the R. fluviale LM-2 genome. Only the top ten GO terms for each category are represented. GO term categories: biological process (green); cellular component (pink); molecular function (blue). The x-axis indicates the number of genes assigned to the same GO term. One unigene may be matched to multiple GO terms. [file 13068_2022_2251_MOESM4_ESM.docx]

**
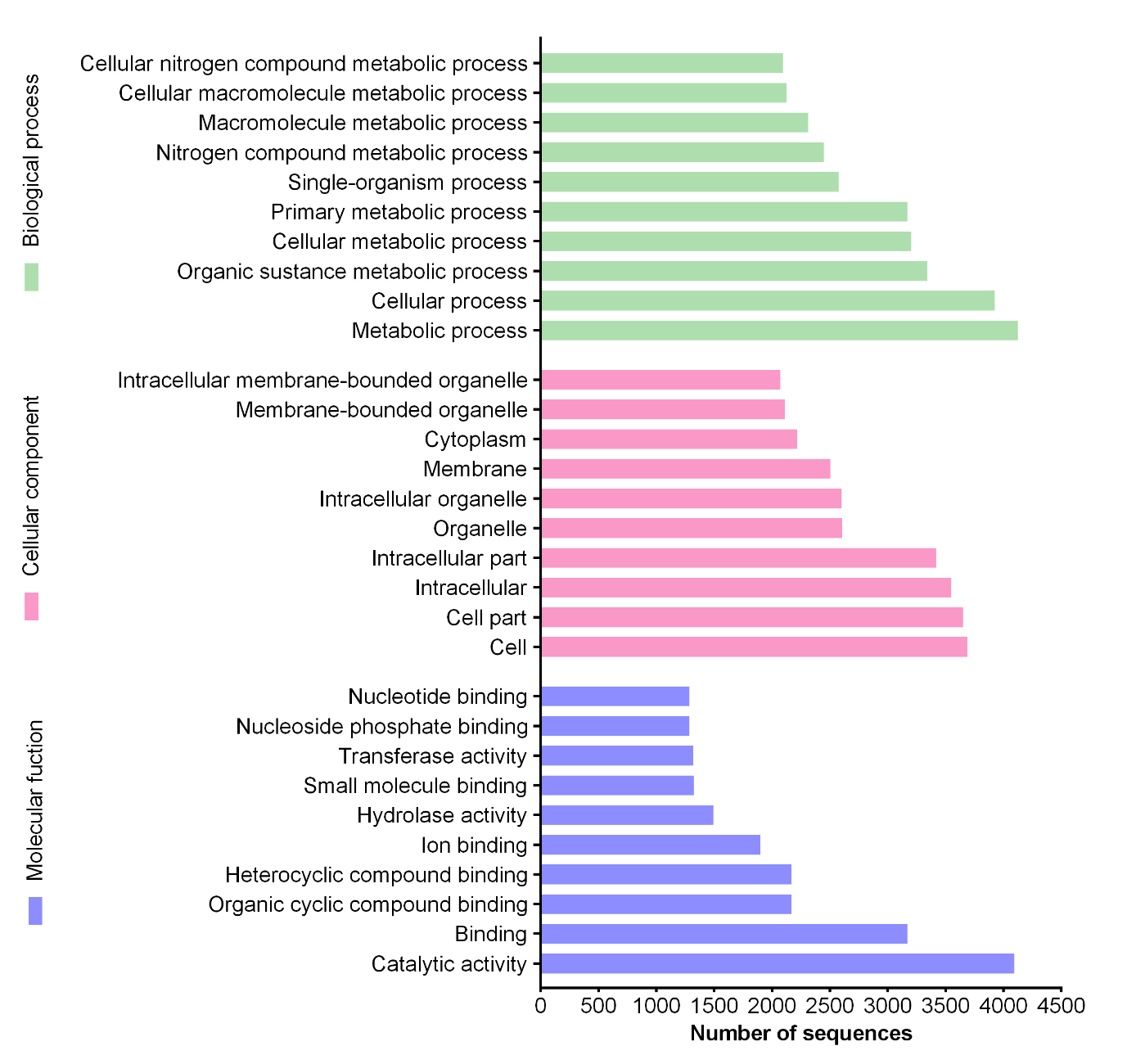
**

**Figure S4. Most abundant Gene ontology (GO) terms assigned to the *R. fluviale* LM-2 genome.** Only the top ten GO terms for each category are represented. GO term categories: biological process (green); cellular component (pink); molecular function (blue). The x-axis indicates the number of genes assigned to the same GO term. One unigene may be matched to multiple GO terms
