# Supplementary material for: Integrative omics analyses of the ligninolytic Rhodosporidium fluviale LM-2 disclose catabolic pathways for biobased chemical production
Source: Biotechnol Biofuels Bioprod. 2023 Jan 9;16:5. doi: 10.1186/s13068-022-02251-6 (PMC9830802; doi:10.1186/s13068-022-02251-6)
Supplement: Supplementary file 5 — Additional file 5: Figure S5. Outline of ferulic acid catabolism by R. fluviale LM-2. Based on genomic analysis, two catabolic pathways were predicted for ferulic acid bioconversion. A) Ferulic acid pathway I: ferulic acid conversion into vanillin in two steps with ATP consumption: i) CoA-thioesterification of ferulic acid by ferulic acid synthetase (FCS); ii) hydration of feruloyl-CoA by ferulic acid hydratase lyase (FCHL). B) Ferulic acid pathway II: cofactor-independent ferulic acid decarboxylation into 4-vinyl guaiacol (4-VG) by a phenolic acid decarboxylase (PDC). [file 13068_2022_2251_MOESM5_ESM.docx]

**
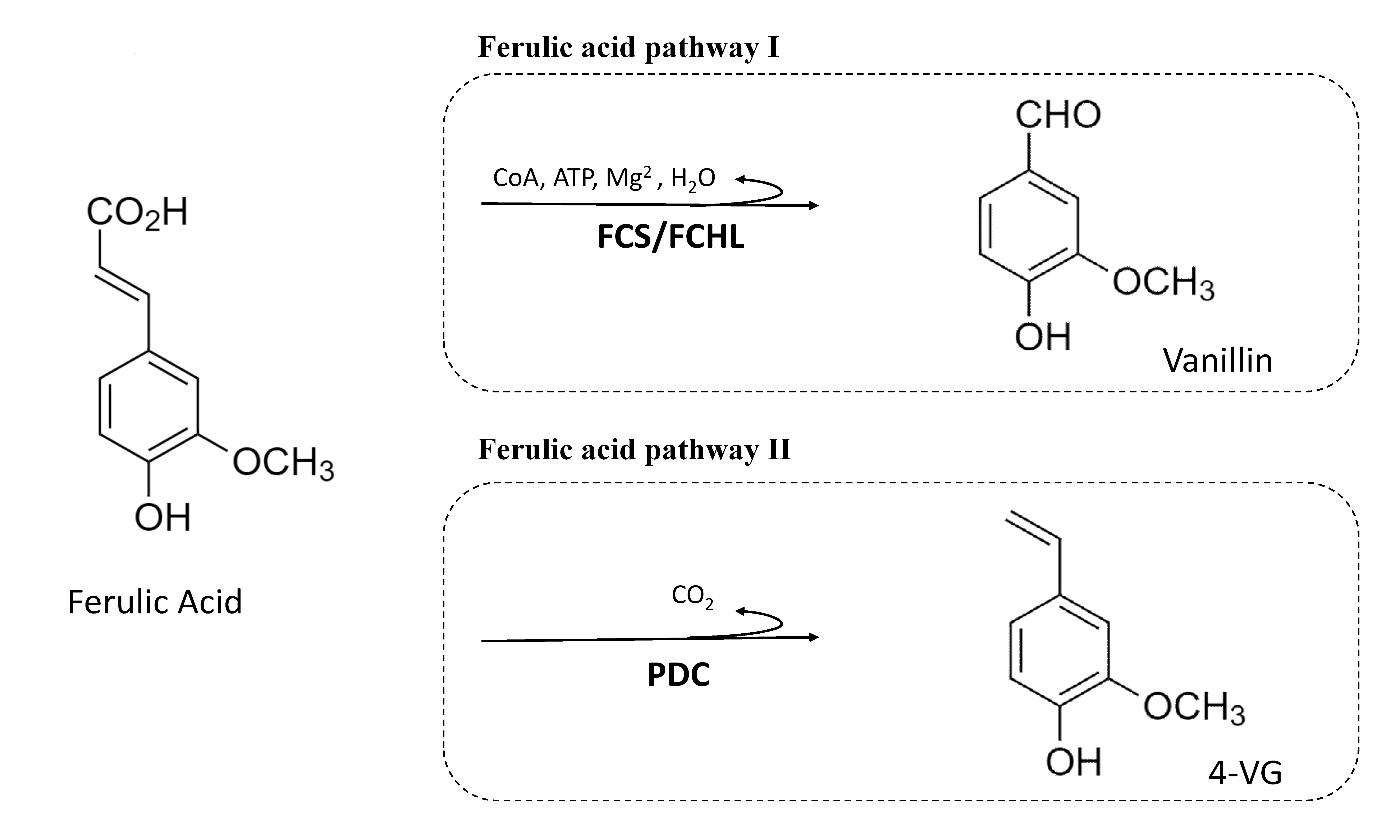
**

**Figure S5.** **Outline of ferulic acid catabolism by *R. fluviale* LM-2.** Based on genomic analysis, two catabolic pathways were predicted for ferulic acid bioconversion. A) Ferulic acid pathway I: ferulic acid conversion into vanillin in two steps with ATP consumption: i) CoA-thioesterification of ferulic acid by ferulic acid synthetase (FCS); ii) hydration of feruloyl-CoA by ferulic acid hydratase lyase (FCHL). B) Ferulic acid pathway II: cofactor-independent ferulic acid decarboxylation into 4-vinyl guaiacol (4-VG) by a phenolic acid decarboxylase (PDC).
