# Supplementary material for: Integrative omics analyses of the ligninolytic Rhodosporidium fluviale LM-2 disclose catabolic pathways for biobased chemical production
Source: Biotechnol Biofuels Bioprod. 2023 Jan 9;16:5. doi: 10.1186/s13068-022-02251-6 (PMC9830802; doi:10.1186/s13068-022-02251-6)
Supplement: Supplementary file 6 — Additional file 6: Figure S6. Differential gene expression analysis of R. fluviale LM-2 in response to kraft lignin. A) Volcano plot. X-axis: Log2 fold change (with lignin/without lignin). Y-axis: negative log10-adjusted p value. Red data points indicate upregulated transcripts, green data points indicate downregulated transcripts, and gray data points indicate nonmodulated genes. B) Number of up- and downregulated and nonmodulated genes. [file 13068_2022_2251_MOESM6_ESM.docx]

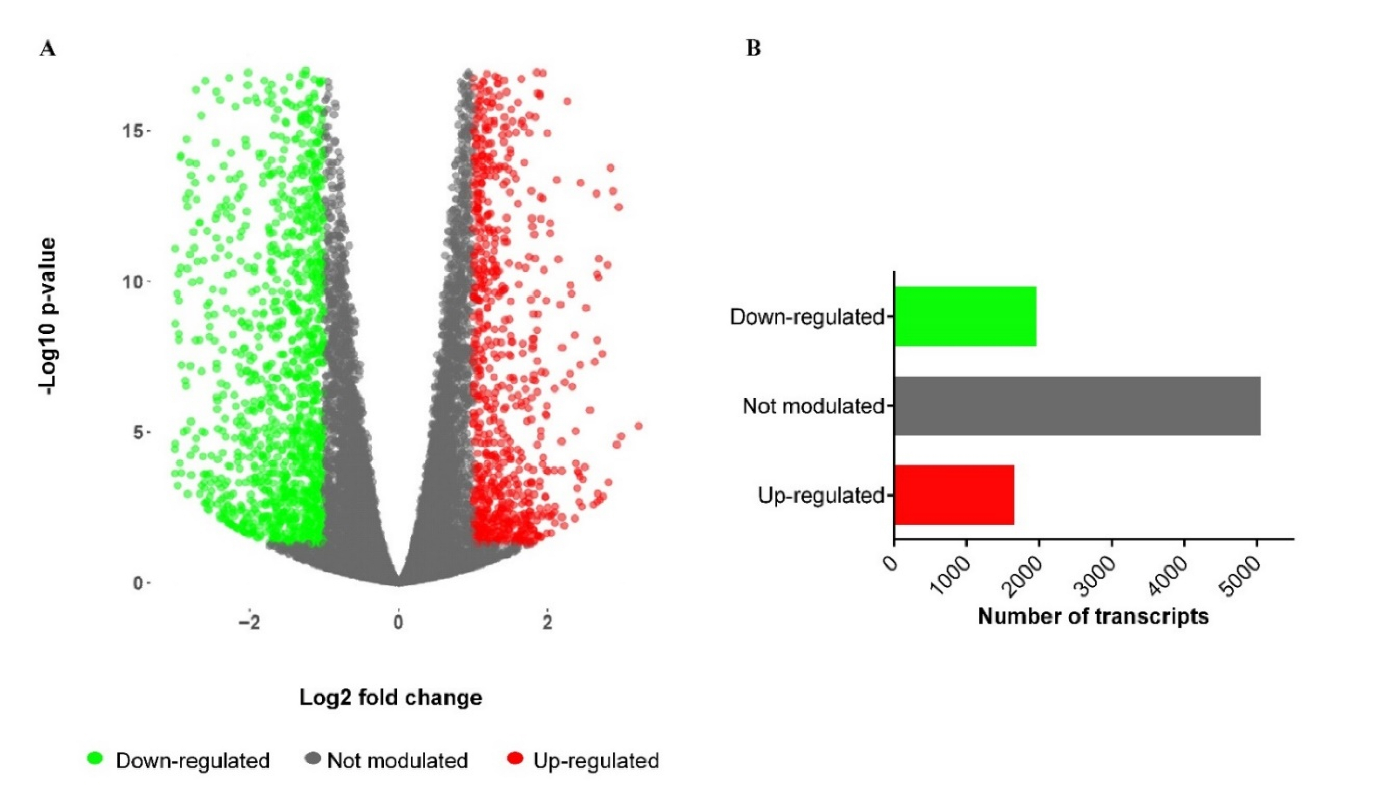


**Figure S6. Differential gene expression analysis of *R. fluviale* LM-2 in response to kraft lignin.** A) Volcano plot. X-axis: Log_2_ fold change (with lignin/without lignin). Y-axis: negative log10-adjusted p value. Red data points indicate upregulated transcripts, green data points indicate downregulated transcripts, and gray data points indicate nonmodulated genes. B) Number of up- and downregulated and nonmodulated genes.
