# Supplementary material for: Integrative omics analyses of the ligninolytic Rhodosporidium fluviale LM-2 disclose catabolic pathways for biobased chemical production
Source: Biotechnol Biofuels Bioprod. 2023 Jan 9;16:5. doi: 10.1186/s13068-022-02251-6 (PMC9830802; doi:10.1186/s13068-022-02251-6)
Supplement: Supplementary file 7 — Additional file 7: Table S1. Genomic reads details. [file 13068_2022_2251_MOESM7_ESM.docx]

**Table S1. Genomic reads details.**

| **Sample** | **Input** | **Both surviving** | **Forward only** | **Reverse only** | **Dropped** |
| --- | --- | --- | --- | --- | --- |
| ***R. fluviale* LM-2 Pair-End** | 9,839,814 | 7,368,112 (74.88%) | 1,509,000 (15.34%) | 193,770 (1.97%) | 768,932 (7.81%) |
| ***R. fluviale* LM-2 Mate-Pair** | 5,873,616 | 1757424 (29.92%) | 1,995,413 (33.97%) | 1,440,090 (24.52%) | 680,689 (11.59%) |

Nextera and Nextera Mate pair libraries were sequenced at Miseq using 2×300 pb.
