# Supplementary material for: Integrative omics analyses of the ligninolytic Rhodosporidium fluviale LM-2 disclose catabolic pathways for biobased chemical production
Source: Biotechnol Biofuels Bioprod. 2023 Jan 9;16:5. doi: 10.1186/s13068-022-02251-6 (PMC9830802; doi:10.1186/s13068-022-02251-6)
Supplement: Supplementary file 9 — Additional file 9: Table S3. Top 10 up- and downregulated genes of R. fluviale LM-2. [file 13068_2022_2251_MOESM9_ESM.docx]

**Table S3. Top 10 up- and downregulated genes of *R. fluviale* LM-2.**

| **Gene ID** | **Log_2_ fold change** | **Padj** | **Description** |
| --- | --- | --- | --- |
| **Upregulated** |  |  |  |
| RF_11942 | 8,563716 | 6,9E-173 | hypothetical protein |
| RF_16583 | 8,327086 | 0 | hypothetical protein |
| RF_03690 | 7,900018 | 0 | hypothetical protein |
| RF_04016 | 7,754333 | 9,1E-149 | DHN family protein |
| RF_12093 | 7,344954 | 2,36E-88 | BTB/POZ and TAZ domain-containing protein 3 |
| RF_12102 | 6,796701 | 3,05E-91 | hypothetical protein |
| RF_03285 | 6,56288 | 1,6E-281 | cation binding protein |
| RF_02870 | 6,461141 | 8,7E-187 | hypothetical protein |
| RF_17046 | 6,258618 | 0 | cation binding protein |
| RF_11611 | 6,227273 | 9,9E-131 | hypothetical protein |
|  |  |  |  |
| **Downregulated** |  |  |  |
| RF_07489 | -9,10122 | 2,7E-89 | Fructose facilitator (MFS transporter) |
| RF_12832 | -8,83709 | 1,4E-173 | Expansin family protein |
| RF_07479 | -8,35988 | 1,5E-283 | Fructose facilitator (MFS transporter) |
| RF_03901 | -8,16409 | 1,2E-303 | MFS general substrate transporter |
| RF_08565 | -8,02041 | 1,2E-135 | Expansin family protein |
| RF_07487 | -7,76153 | 3,1E-175 | Fructose facilitator (MFS transporter) |
| RF_11374 | -7,6533 | 4,5E-120 | Zinc/iron permease |
| RF_15230 | -7,57818 | 3,21E-86 | Nucleoside-diphosphate-sugar epimerase |
| RF_06445 | -7,55557 | 0 | MFS general substrate transporter |
| RF_17165 | -7,44534 | 2,36E-38 | Purine-cytosine permease |

The classification was based on the log2 fold change value. Padj is the adjusted p value (≥ 0.05).
