# Supplementary material for: Integrative omics analyses of the ligninolytic Rhodosporidium fluviale LM-2 disclose catabolic pathways for biobased chemical production
Source: Biotechnol Biofuels Bioprod. 2023 Jan 9;16:5. doi: 10.1186/s13068-022-02251-6 (PMC9830802; doi:10.1186/s13068-022-02251-6)
Supplement: Supplementary file 10 — Additional file 10: Table S4. Phenolic compounds detected by UHPLC–MS/MS analysis. [file 13068_2022_2251_MOESM10_ESM.docx]

**Table S4. Phenolic compounds detected by UHPLC–MS/MS analysis.**

| **Compound** | **Chemical**  **structure** | **Formula [M+H]^+^** | **Theoretical**  **(*m/z*)** | **Experimental (*m/z*)** | **Time**  **(min)** | **Error**  **(ppm)** | **MS/MS**  **Product Ions** | **Reference** |
| --- | --- | --- | --- | --- | --- | --- | --- | --- |
| **Vanillin** |  | C_8_H_9_O_3_ | 153.0546 | 153.0546 | 4.72 | 0.06 | 153.0547, **125.0598,** 111.0444, 93.0338, 71.9408, 65.0393 | (83) |
| **Ferulic acid** |  | C_10_H_11_O_4_ | 195.0652 | 195.0652 | 5.63 | 0.18 | 195.0650, **177.0546**, 145.0284, 134.0361, 117.0338, 89.0391 | (84) |
| **4-Vinylguaiacol** |  | C_9_H_11_O_2_ | 151.0754 | 151.0754 | 4.71 | 0.03 | **151.0754**, 133.1012, 119.0493, 107.0858, 91.0547, 81.0702 | (85) |
